# Supplementary material for: Retroviral Retention Activates a Syk-Dependent HemITAM in Human Tetherin
Source: Cell Host Microbe. 2014 Sep 10;16(3):291–303. doi: 10.1016/j.chom.2014.08.005 (PMC4161388; doi:10.1016/j.chom.2014.08.005)
Supplement: Document S1. Supplemental Experimental Procedures and Figures S1–S6 [file mmc1.pdf]

Cell Host & Microbe, Volume *16*

Supplemental Information

Retroviral Retention Activates a Syk-Dependent HemITAM in Human Tetherin

Rui Pedro Galão, Suzanne Pickering, Rachel Curnock, and Stuart J.D. Neil

Supplemental Information

Supplemental Figure Inventory

Figure S1 (related to Figure 1): Transient co-expression of tetherin mutants Y6A or Y8A have a dominant-negative effect on tyrosine phosphorylation of wild-type tetherin

Figure S2 (related to Figure 2): Tetherin phosphorylation induced by retroviral assembly.

Figure S3 (related to figure 4): Treatment of cells with Tyrosine Kinase inhibitors or Syk siRNA depletion.

Figure S4 (related to Figure 5): Multiple context-dependent adaptations have resulted in the signaling capacity of human tetherin.

Figure S5 (related to Figure 6): Cytochalasin D treatment does not affect tetherin surface expression, and tyrosine phosphorylation of tetherin occurs preferentially in detergent resistant membranes.

Figure S6 (related to Figure 7): Tetherin R19H polymorphism is defective for tetherin-mediated NFkB activation but not virion retention.

Supplemental Figures

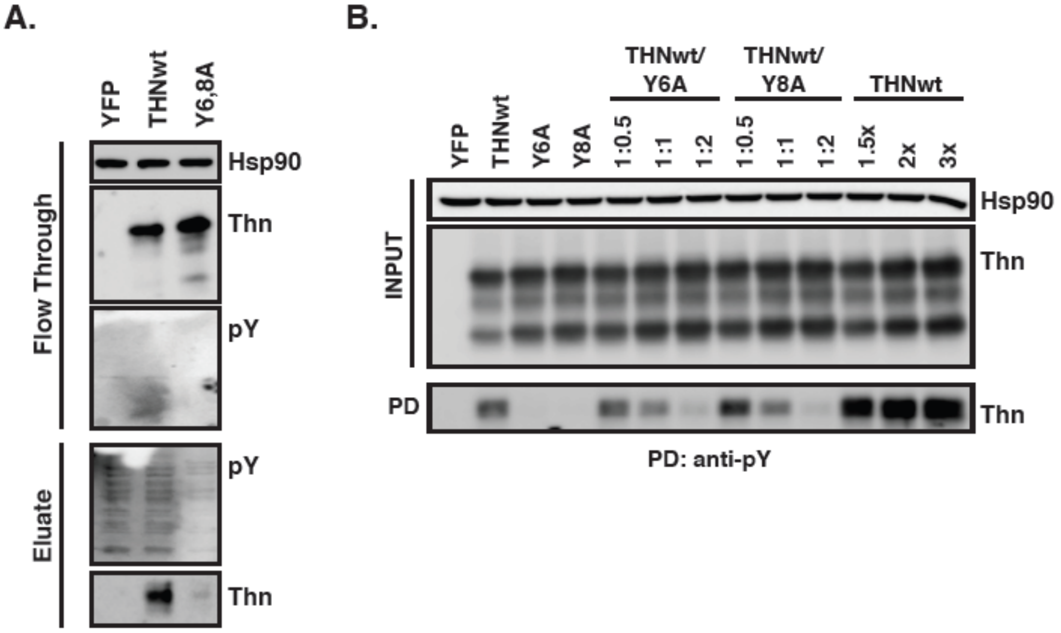

Figure S1 (related to Figure 1). Transient co-expression of tetherin mutants Y6A or Y8A have a dominant-negative effect on tyrosine phosphorylation of wild-type tetherin

(A) Phosphorylated proteins were isolated from lysates of 293 cells transiently expressing wild-type tetherin, double tyrosine mutant (Y6,8A) or YFP as control, by using a Phosphoprotein Purification Column. Phosphorylation status of the proteins was assessed by western blot analysis of the flow-through and eluated fractions. (B) Lysates from 293 cells co-transfected with a fixed amount of wild type tetherin plus increasing amounts of Y6A or Y8A, were immunoprecipitated with an anti-phosphotyrosine antibody and precipitates analyzed by western blot for the presence of tetherin.

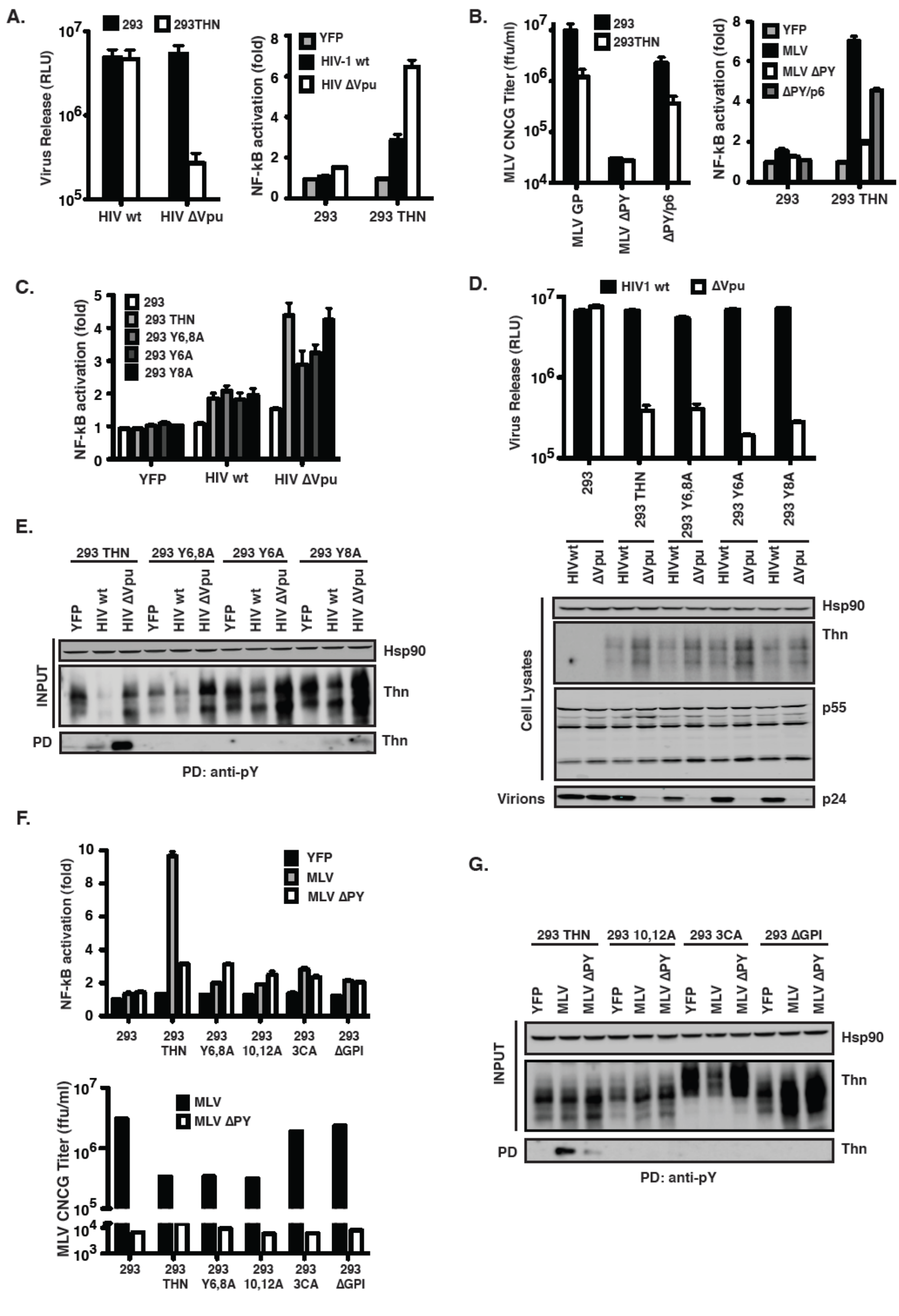

Figure S2 (related to Figure 2). Tetherin phosphorylation induced by retroviral assembly.

(A, B) Infectious virus release from supernatants of Figure 2A was determined on HeLa-TZMbl indicator cells (A, left panel), or by titration of MLV

supernatants on 293T cells and analyzed by flow cytometry for GFP expression 48h later (B, left panel). NF-kB reporter fold activation in 293 and 293/tetherin cells transfected with HIV-1 wt or HIV-1 ΔVpu (A, right panel), or MLV provirus or derivatives (MLVΔPY, MLVΔPY/p6) (B, right panel). **(C)** NF-kB reporter fold activation in 293 cells stably expressing wild-type tetherin, the double tyrosine mutant (Y6,8A) or the single tyrosine mutants (Y6A or Y8A) transfected with HIV-1 wild-type or HIV-1 ΔVpu proviruses. **(D)** Infectious virus release from C was determined as in (A) and physical particle yield analyzed by western blot of cell lysates and pelleted supernatants using an anti-p24 monoclonal antibody. **(E)** Lysates from (C) were used for immunoprecipitated with anti-pY antibody. Pulled-down fractions were analyzed for tetherin co-precipitation by western blot. **(F)** Stable cell lines expressing wild-type tetherin, the Y6,8A or RVP(10-12)AAA mutants or the structural mutants 3CA (cysteine-less), or ΔGPI (truncated form lacking the GPI anchor) were assayed for their ability to activate NFkB luciferase reporter upon transfection with MLV provirus or the MLVΔPY derivative (upper panel). MLV virus titers were assayed as in (B) (lower panel). **(G)** Lysates from (F) were immunprecipitated with anti-pY, deglycosylated and blotted for tetherin. NFkB luciferase reporter fold changes relative to 293 cells transfected with YFP control (A, B, C and F). All error bars represent ±SEM of three experiments.

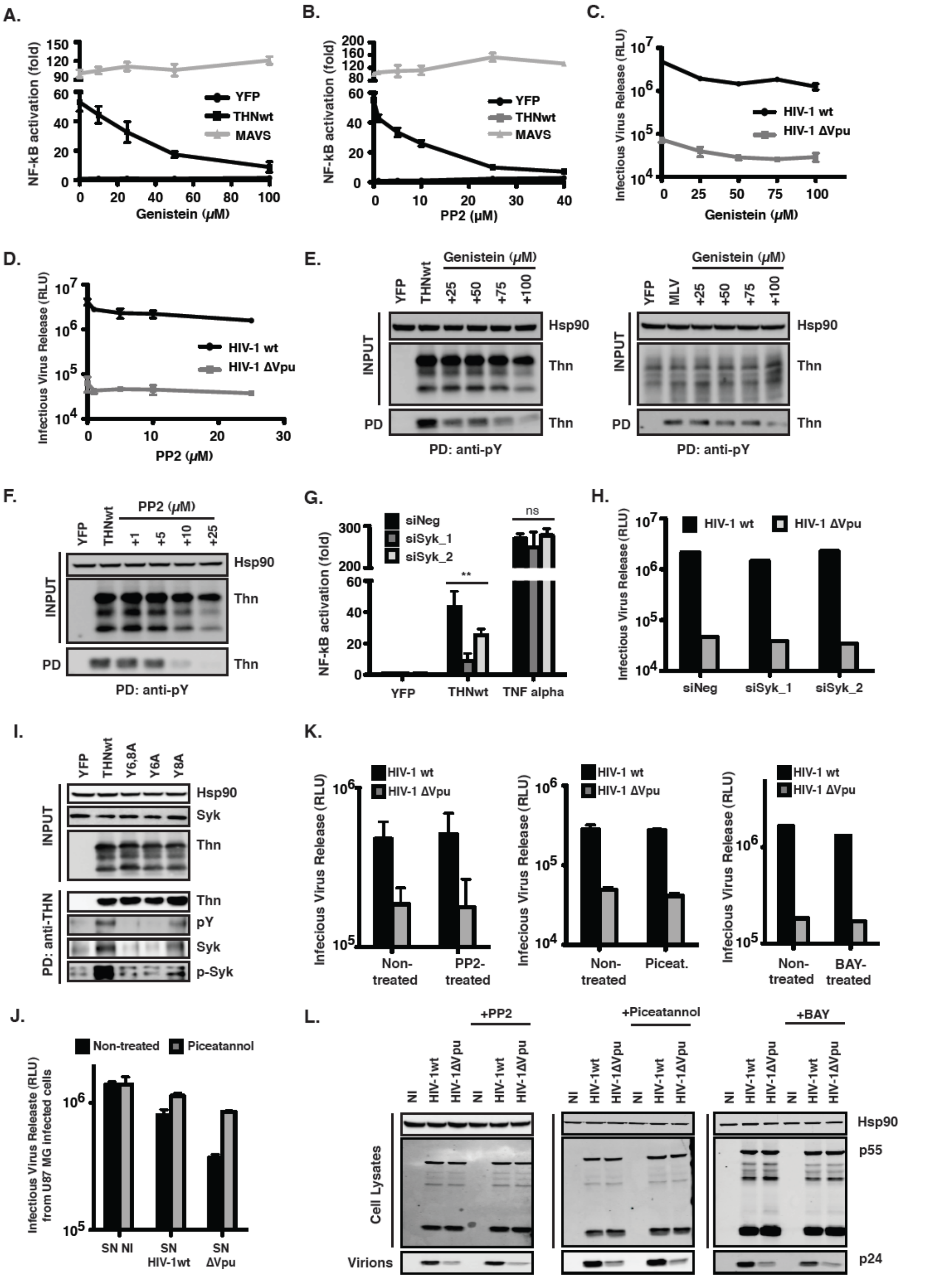

Figure S3 (related to figure 4). Treatment of cells with Tyrosine Kinase inhibitors or Syk siRNA depletion.

(A, B) NF-kB reporter fold activation in 293 cells transiently expressing tetherin, or MAVS as control, were treated with increasing amounts of the

inhibitors Genistein (A) or PP2 (B). Fold changes relative to cells transfected with YFP and non-treated with inhibitors. **(C, D)** HIV-1 Infectious viral particle release from supernatants from Figure 4A (C) and Figure 4B (D) were determined on HeLa-TZMbl indicator cells. **(E, F)** Lysates from parallel experiments to Figure S3A (E, left), Figure 4A (E, right) and Figure S3B (F) were immunoprecipitated with an anti-phosphotyrosine antibody and precipitates analyzed as before **(G)** 293 cells were transfected twice over 72h with siRNAs directed against Syk (siSyk_1 and _2) or a non-targeting control (siNeg). Cells were then transiently transfected with the NFκB reporter plasmid either with tetherin or treated with TNFα (1ng/ml). Fold increases in luciferase expression are plotted relative to YFP control. All error bars represent ±SEM of three experiments. *$p > 0.05$, **$p > 0.01$, and ***$p > 0.001$ as determined by paired two-tailed t test. **(H)** HIV-1 Infectious viral particle release from supernatants from Figure 4D were determined as in (C, D). **(I)** Lysates of 293 cells transiently expressing wild type tetherin or tyrosine mutants were immunoprecipitated with anti-tetherin antibodies. Pull-downs were analyzed by western blot for tetherin, phosphotyrosine, Syk and its active phosphorylated form (pSyk). **(J)** U87MG cells were treated overnight with supernatants from non-treated or Piceatannol-treated CD4+ve T cells infected in Figure 4J, and then challenged with VSV-G pseudotyped HIV-1 wt virus (MOI of 0.5) in a one-round viral replication assay. Infectious virus release was determined 48 hours later as in (C, D). **(K, L)** Infectious virus release from cells in Figure 4J was determined by infection of HeLa-TZMbl reporter cell lines (K) and western blot analysis of cell lysates and supernatants for HIV-1 p24-CA (L).

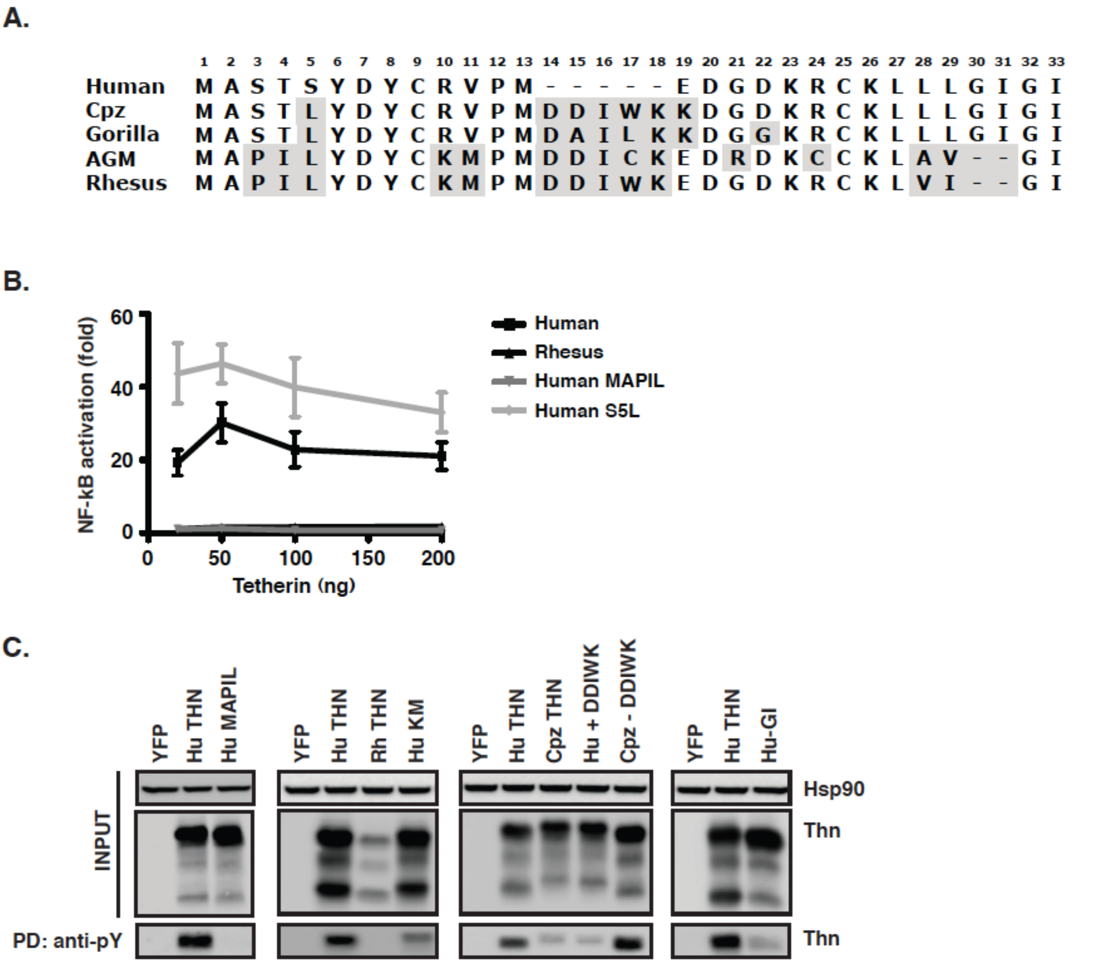

Figure S4 (related to Figure 5). Multiple context-dependent adaptations have resulted in the signaling capacity of human tetherin.

(A) Alignment of cytoplasmic tails from different primate tetherins with segments differing from human tetherin highlighted in gray. **(B)** NFkB Luciferase reporter activation in 293 cells transiently transfected with increasing amounts of human tetherins bearing rhesus-specific mutation in positions 3-4. All error bars represent ±SEM of three experiments. **(C)** Anti-pY immunoprecipitates of 293 cells transiently transfected with the indicated tetherin mutant were deglycosylated and analyzed for tetherin pulldown by western blot.

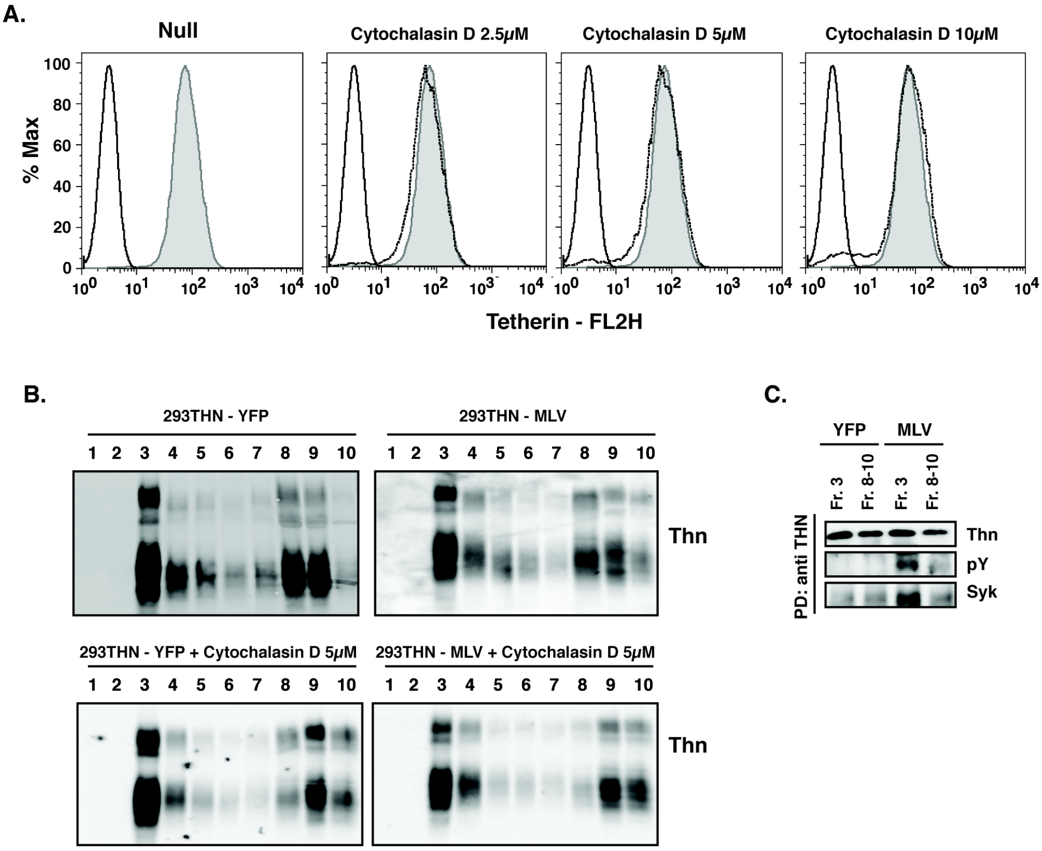

Figure S5 (related to Figure 6). Cytochalasin D treatment does not affect tetherin surface expression, and tyrosine phosphorylation of tetherin occurs preferentially in detergent resistant membranes.

(A) 293/tetherin cells were treated with increasing amounts of cytochalasin D for 8 hours and tetherin expression and the cell surface was determined by flow cytometry using anti-human tetherin-PE antibody. **(B)** 293/tetherin cells transfected with YFP or MLV provirus were mock- or cytochalasin D-treated (5μM) for 6 hours and then lysed and DRMs separated on an iodixinol gradient. Localization of tetherin was determined by western blotting the different indicated gradient fractions. **(C)** Tetherin was immunoprecipitated from DRM (fraction 3) or soluble (Fractions 8-10) fractions and pulled-downs were western blotted for tyrosine phosphorylated tetherin (anti-pY) and Syk.

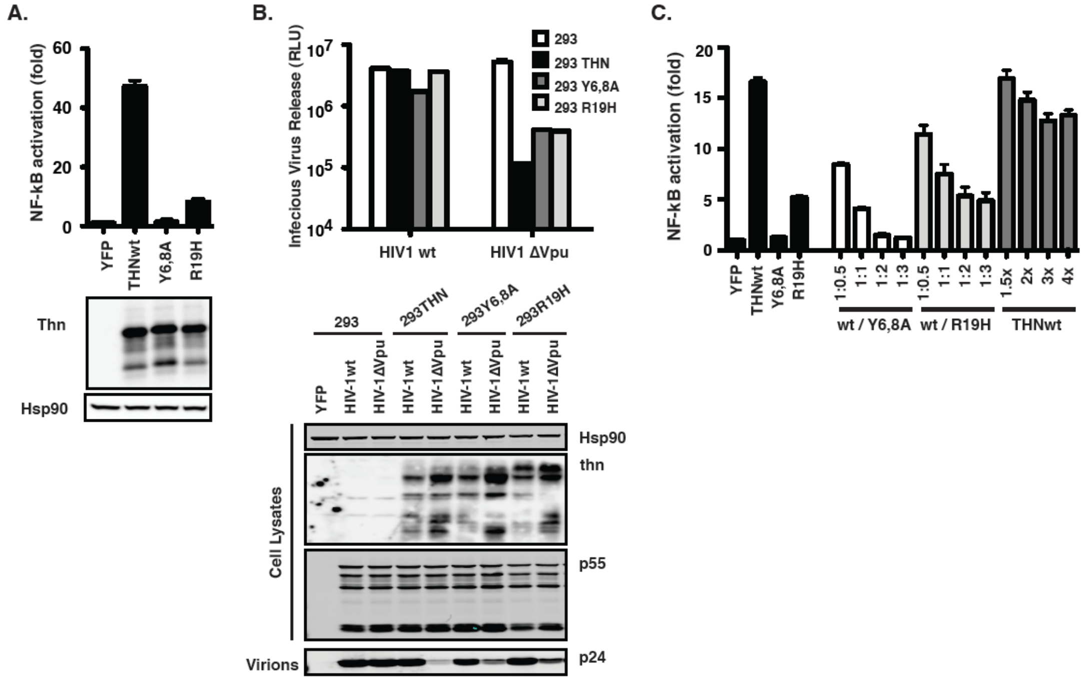

Figure S6 (related to Figure 7). Tetherin R19H polymorphism is defective for tetherin-mediated NFkB activation but not virion retention.

(A) Fold activation of a NFkB reporter in 293 cells transiently transfected with wild-type tetherin, double-tyrosine mutant (Y6,8A) or the human CT polymorphism R19H, compared to control YFP vector (upper). Tetherin expression levels were confirmed by western blot (lower). **(B)** Infectious virus release from supernatants from Figure 7B was determined on HeLa-TZMbl indicator cells (upper) and western blot analysis of cell lysates and supernatants for HIV-1 p24-CA (lower). **(C)** Fold activation of NFkB reporter in 293 cells transiently transfected with fixed amounts of NFkB luciferase reporter and wild-type tetherin plus increasing amounts of tetherin double tyrosine mutant or R19H CT polymorphism (or wild-type as internal control). Total DNA amounts transfected were kept constant by addition of pCR3.1 YFP. Fold changes relative to YFP control. Error bars represent ±SEM of 3 independent experiments.

Supplemental Experimental Procedures

Plasmids and Cells

Tetherin mutants and species orthologs were made in pCR3.1 by standard molecular biology methods. Chimpanzee and Gorilla tetherins were provided by, respectively, G. Towers (UCL) and F. Kirchhoff (Univ. of Ulm). The NFkB-dependent 3xkB-pCONA-FLuc reporter construct and the CMV-RLuc control plasmid were provided by A. MacDonald (Mankouri et al., 2010), while the MAVS expression vector was provided by J. Luban (UMASS). The molecular clones of HIV-1 NL4.3 and derived ΔVpu mutant have been described previously (Neil et al., 2006). pMLV NCS (MLV), pMLV NCS ΔPY and pMLVp6pY plasmids were provided by J. Martin-Serrano (KCL) (Martin-Serrano et al., 2004). A codon optimized RICH2 cDNA was synthesized by MWG and subcloned into pCR3.1 with an N-terminal HA tag using standard procedures. Human Embryonic Kidney 293 (HEK-293), 293T (HEK-293T) and U87-MG cells were obtained from the ATCC, while the HeLa TZM indicator cell line was obtained from Dr. John C. Kappes through the NIH AIDS Reagent Repository Program (ARRP). All adherent cells were maintained in DMEM supplemented with 10% fetal calf serum (FCS) and gentamicin.

To produce 293 transductants stably expressing tetherin mutants, the indicated mutant was cloned into the retroviral vector pLHCX (Clontech). Retroviral vector stocks were produced by cotransfection of 293T cells with MLV Gag-Pol expression vector, pMD.G (VSV-G) and pCR3.1-Vpu. 293 cells were transduced 48h later and hygromycin resistant populations were selected. Derivatives stably expressing some of the human tetherin mutants have been described previously (Galao et al., 2012).

Antibodies

Cell lysates, IPs and eluates were subjected to SDS-PAGE and western blots performed using the following antibodies: rabbit anti-BST2 (NIH ARRP), mouse anti-BST2 monoclonal (clone 3H4) (Abnova), rabbit anti-phosphotyrosine (Calbiochem), mouse anti-phosphotyrosine monoclonal (clone 4G10) (Millipore), mouse anti-HA.11 (Covance); from Cell Signaling - rabbit monoclonal anti-Syk (D1I5Q), rabbit anti-phospho Syk (Y323), rabbit anti-ZAP70, rabbit anti-phospho ZAP70 (Y292), rabbit anti-TAK1 and rabbit anti-TRAF2; from Santa Cruz Biotechnologies: rabbit anti-hsp90 α/ß, goat anti-RICH2 (S-16) and rabbit anti-TRAF6 (H-274). For HIV-1 Gag/p24, hybridoma supernatant of monoclonal ab 183 (NIH ARRP) was used at 1:100. Visualization was performed by ImageQuant using anti-mouse or anti-rabbit HRP-linked antibodies (NEB, UK) or an anti-goat-HRP linked antibody (Santa Cruz Biotechnologies), or on a LiCor imager using goat-anti mouse IRD-680/goat-anti rabbit IRD800 conjugated secondary antibodies (LiCor).

References

Galao, R.P., Le Tortorec, A., Pickering, S., Kueck, T., and Neil, S.J. (2012). Innate sensing of HIV-1 assembly by Tetherin induces NFkappaB-dependent proinflammatory responses. Cell Host Microbe *12*, 633-644.

Mankouri, J., Fragkoudis, R., Richards, K.H., Wetherill, L.F., Harris, M., Kohl, A., Elliott, R.M., and Macdonald, A. (2010). Optineurin negatively regulates the induction of IFNbeta in response to RNA virus infection. PLoS Pathog *6*, e1000778.

Martin-Serrano, J., Perez-Caballero, D., and Bieniasz, P.D. (2004). Context-dependent effects of L domains and ubiquitination on viral budding. J Virol *78*, 5554-5563.

Neil, S.J., Eastman, S.W., Jouvenet, N., and Bieniasz, P.D. (2006). HIV-1 Vpu promotes release and prevents endocytosis of nascent retrovirus particles from the plasma membrane. PLoS Pathog *2*, e39.
