# Supplementary material for: Retroviral Retention Activates a Syk-Dependent HemITAM in Human Tetherin
Source: Cell Host Microbe. 2014 Sep 10;16(3):291–303. doi: 10.1016/j.chom.2014.08.005 (PMC4161388; doi:10.1016/j.chom.2014.08.005)
Supplement: Document S2. Article plus Supplemental Information [file mmc2.pdf]

CellPress

Retroviral Retention Activates a Syk-Dependent HemITAM in Human Tetherin

Rui Pedro Galão,[1] Suzanne Pickering,[1] Rachel Curnock,[2] and Stuart J.D. Neil[1,*]
[1]Department of Infectious Disease, King's College London School of Medicine, Guy's Hospital, London SE1 9RT, UK
[2]School of Biochemistry, Faculty of Medical and Veterinary Sciences, University of Bristol, Bristol BS8 1TD, UK
*Correspondence: stuart.neil@kcl.ac.uk
http://dx.doi.org/10.1016/j.chom.2014.08.005
This is an open access article under the CC BY license (http://creativecommons.org/licenses/by/3.0/).

SUMMARY

Tetherin (BST2/CD317) restricts the release of enveloped viral particles from infected cells. Coupled to this virion retention, hominid tetherins induce proinflammatory gene expression via activating NF-κB. We investigated the events initiating this tetherin-induced signaling and show that physical retention of retroviral particles induces the phosphorylation of conserved tyrosine residues in the cytoplasmic tails of tetherin dimers. This phosphorylation induces the recruitment of spleen tyrosine kinase (Syk), which is required for downstream NF-κB activation, indicating that the tetherin cytoplasmic tail resembles the hemi-immunoreceptor tyrosine-based activation motifs (hemITAMs) found in C-type lectin pattern recognition receptors. Retroviral-induced tetherin signaling is coupled to the cortical actin cytoskeleton via the Rac-GAP-containing protein RICH2 (ARHGAP44), and a naturally occurring tetherin polymorphism with reduced RICH2 binding exhibits decreased phosphorylation and NF-κB activation. Thus, upon virion retention, this linkage to the actin cytoskeleton likely triggers tetherin phosphorylation and subsequent signal transduction to induce an antiviral state.

INTRODUCTION

Tetherin (bone marrow stromal cell antigen 2 [BST2] or CD317) is an interferon-induced membrane protein that inhibits the release of diverse enveloped viral particles from infected cells (reviewed in Neil, 2013). Topologically, tetherin consists of a short N-terminal cytoplasmic tail (CT), a transmembrane (TM) domain, an extracellular rod-like coiled coil, and a C-terminal GPI anchor. Parallel tetherin dimers partition into budding virions such that after membrane scission, the GPI anchors of tetherin are predominantly retained in the viral membrane (Perez-Caballero et al., 2009; Venkatesh and Bieniasz, 2013). Retained virions can be endocytosed and targeted for endosomal degradation (Neil et al., 2006; Neil et al., 2008). There are now several examples of virally encoded countermeasures that target tetherin. These include the accessory proteins Nef and Vpu of primate and human lentiviruses, respectively, which target the tetherin orthologs of their host species (Neil, 2013), and numerous lines of evidence indicate that this function is maintained and selected for throughout infection and upon cross-species transmission (Götz et al., 2012; Pickering et al., 2014; Sauter et al., 2012; Serra-Moreno et al., 2011). It is likely that the adaptation of Vpu to target human tetherin was a key event in the spread of HIV-1 group M to become the predominant agent of the HIV/AIDS pandemic (Sauter et al., 2009).

Alongside the strong genetic evidence that tetherin targets primate lentiviruses in vivo, studies in mice indicate that tetherin modulates retroviral pathogenesis (Barrett et al., 2012; Liberatore and Bieniasz, 2011). Physical restriction of virion release also has further associated antiviral effects. Recent studies have shown that CD4+ T cells infected with HIV-1 mutants lacking Vpu are more sensitive to antibody-dependent cellular cytoxicity (ADCC) (Arias et al., 2014; Veillette et al., 2014). This is in part due to enhanced opsonization of tetherin-retained virions by antibodies targeting the viral envelope glycoprotein. Additionally, we and others have shown that human tetherin is a potent activator of NF-κB when it restricts the release of retroviral and filoviral particles (Cocka and Bates, 2012; Galão et al., 2012; Tokarev et al., 2013). This is determined by the recruitment of the E3 ubiquitin ligases TRAF2 and TRAF6 that mediate the activation of the kinase TAK1 (Galão et al., 2012; Tokarev et al., 2013). In keeping with this, there is an increase in the secretion of proinflammatory cytokines from primary human CD4+ T cells infected with Vpu-defective HIV-1 that is tetherin dependent (Galão et al., 2012). These observations suggest that the coupling of proinflammatory signaling to tetherin's antiviral activity allows it to act as a sensor of viral assembly, making the infected cell more visible to systemic innate and adaptive immunity.

Mechanistically, tetherin-mediated signal transduction requires both the structural attributes essential for restriction and sequences in the CT (Galão et al., 2012; Tokarev et al., 2013). Among these is a dual-tyrosine-based motif (YDYCRV), previously shown to act as an endocytic sorting signal (Rollason et al., 2007), that promotes the recycling of tetherin to and from the cell surface. In the absence of this sequence, the TRAF/TAK1 complex does not interact with tetherin (Galão et al., 2012; Tokarev et al., 2013). However, blockade of virion endocytosis from the surface potentiates rather than abolishes signaling, suggesting tetherin clustering in assembling virions as the primary trigger (Galão et al., 2012). Although the tyrosine residues are well conserved, the ability of mammalian tetherins to signal in human cells is highly species specific. Amino acid changes in the tetherin CT that occurred during hominoid

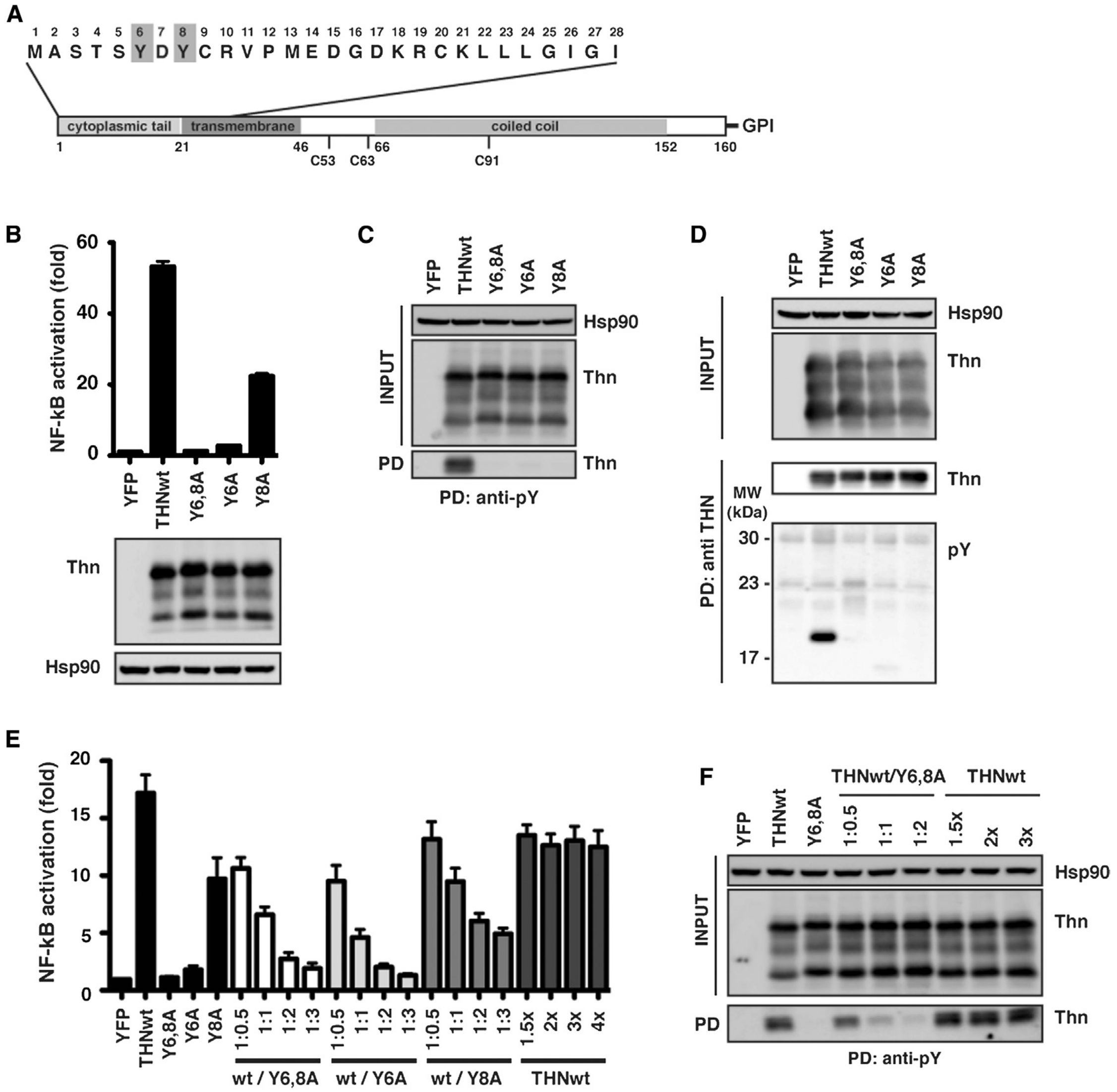

Figure 1. Tyrosine Phosphorylation of Transiently Expressed Human Tetherin

(A) Schematic representation of tetherin including the amino acid sequence of the cytoplasmic tail from human tetherin, with the conserved tyrosines highlighted in gray.

(B) Fold activation of a firefly luciferase NF-κB reporter in 293 cells transiently transfected with tetherin, and mutants thereof, compared to control YFP vector (top). Tetherin expression levels were confirmed by western blot (bottom).

(C) Lysates of 293 cells transiently expressing tetherin or the indicated mutant were immunoprecipitated with an anti-pY antibody. Deglycosylated precipitates were analyzed by western blot using an anti-tetherin antibody.

(D) As in (C), but lysates were immunoprecipitated with an anti-tetherin antibody and blotted with an anti-pY antibody.

(E) Fold activation of NF-κB reporter in 293 cells transiently transfected with fixed amounts of NF-κB-luc reporter and wild-type tetherin plus increasing amounts of tetherin tyrosine mutants (or wild-type as internal control). Total DNA amounts transfected were kept constant by the addition of pCR3.1 YFP. Fold changes relative to YFP control.

(F) Lysates from (E) were immunoprecipitated with anti-pY and visualized by western blot. All error bars are ± SEM of three independent experiments. See also Figure S1.

evolution, culminating in a 5 amino acid deletion after divergence from chimpanzees, account for the potency of human tetherin signaling (Galão et al., 2012). Tetherin is also expressed as two isoforms in primary cells (Cocka and Bates, 2012). The shorter of these lacks the tyrosine motif and dominantly inhibits signaling, implying that only homodimers of the long isoform can activate NF-κB. In this study we further characterized the role of this motif in tetherin-mediated signal transduction.

RESULTS

Human Tetherin Is Phosphorylated on Conserved Tyrosines in the CT upon Virion Retention

We have previously shown that human tetherin's signaling is dependent on a tyrosine motif (YDYCRV) (Figure 1A) (Galão et al., 2012; Rollason et al., 2007). Peptides corresponding to the tetherin CT phosphorylated on either tyrosine have been

found in a phosphoproteome screen (St-Germain et al., 2009). We hypothesized that the juxtaposition of two YDYCRV motifs in a tetherin dimer may act as a noncanonical hemi-immunoreceptor tyrosine-based activation motif (hemITAM). Known hemITAMs consist of single SH2-binding domains, YXX-hydrophobic residue, in the CTs of several dimeric C-type lectins, which recruit SH2-containing tyrosine kinases or phosphatases upon phosphorylation (Suzuki-Inoue et al., 2006). Importantly, both monomers of the dimer require intact tyrosine motifs to function. Mutation of the Y6 to an alanine residue completely abolished the activation of an NF-κB-dependent firefly luciferase reporter gene upon transfection of human tetherin into 293 cells, whereas a Y8A mutation affected the efficiency of signaling (Figure 1B). Immunprecipitations (IPs) of cell lysates with anti-phosphotyrosine (anti-pY) antibody under these transfection conditions specifically pull down wild-type (WT) tetherin but not those bearing tyrosine mutations (Figure 1C), indicating tetherin phosphorylation on one or both tyrosine residues. Consistent with this, parallel IP of tetherin yielded a phosphotyrosine-specific band at the size of deglycosylated WT tetherin only (Figure 1D), as did isolation of WT but not Y6,8A mutant tetherin from cell lysates using a phosphoprotein-specific resin (Figure S1A, available online). Tetherin naturally exists as dimers of long and short isoforms, and the short isoform acts as a dominant inhibitor of reporter gene activation (Cocka and Bates, 2012). We found that transient transfection of tetherin Y6,8A in combination with WT at ratios of 2:1 or above completely inhibited tetherin-mediated reporter gene activity (Figure 1E) and the concomitant ability to IP tetherin with anti-pY antibodies (Figure 1F). In agreement with the reporter gene activities above, the single Y6A mutation also displayed this potent dominant-negative activity, whereas the Y8A mutant was weaker (Figures 1E and S1B). Together these data indicate that tetherin can be tyrosine phosphorylated and that this correlates with NF-κB reporter activation. Furthermore, an intact tyrosine motif must be present on both monomers of the mature protein.

Tetherin expressed stably in cell lines does not constitutively signal, but the restriction of viral particle release triggers NF-κB reporter activation (Galão et al., 2012). 293/tetherin cells were transfected either with HIV-1 (WT) or HIV-1ΔVpu proviral clones or with murine leukemia virus (MLV)-based vectors with or without late-budding domain defects (Martin-Serrano et al., 2004). As expected, infectious release of HIV-1ΔVpu and MLV was reduced by between 10- and 30-fold in the presence of tetherin and led to a 6- to 8-fold induction of the cotransfected NF-κB reporter (Figures S2A and S2B). Consistent with this, IP of the same cell lysates with anti-pY or passing the lysate through a phosphoprotein column yielded evidence of tyrosine-phosphorylated tetherin (Figures 2A and 2B) triggered by virus budding. Mutation of the late-budding domain in MLV Gag (MLVΔPY) abolished tetherin phosphorylation, and restoration of viral assembly by a heterologous late domain (MLVΔPY/p6) recovered it. 293 cells expressing single or double tyrosine point mutations showed that Y6 was essential for both MLV- (Figure 2C) and HIVΔVpu-induced NF-κB activity (Figure S2C). As expected, none of the mutations had any effect on the ability of tetherin to restrict virion release (Figures 2C and S2D). However, mutation of Y6 completely abolished the ability of both MLV and HIVΔVpu particles to promote isolation of phosphorylated tetherin (Figures 2D, 2E and S2E). Interestingly, for virus budding, mutation of Y8 appeared to reduce the efficiency of phospho-tetherin isolation, in keeping with the reporter gene activation data. Thus, phosphorylation of Y6 induced by virion budding is essential for tetherin-mediated signal transduction, and this is potentially stabilized by Y8. This facet is more easily detected when tetherin signaling is activated by its physiological ligand (viral particles) rather than by transient overexpression. Finally, we tested tetherin mutants with signaling defects that either do (tetherin RVP[10-12]AAA, which abolishes the last two amino acids of the potential SH2-binding domain) or do not (tetherin-3CA, which lacks the extracellular cysteine residues, or tetherin-ΔGPI) restrict virion release (Galão et al., 2012; Perez-Caballero et al., 2009) (Figures S2F and S2G). In all cases, the ability of MLV assembly to induce tetherin phosphorylation was lost, indicating virion retention as a prerequisite.

In our previous study, restriction of HIV-1ΔVpu release in primary human target cells resulted in proinflammatory cytokine release (Galão et al., 2012). We therefore asked whether we could detect tetherin phosphorylation in infected CD4+ T cells. Purified and activated CD4+ T cells from two donors were infected with HIV-1 (WT) or HIV-1ΔVpu at a multiplicity of infection (moi) of 3 to ensure >90% infection. After 48 hr, viral release was assessed, and as expected, infectious HIV-1ΔVpu release was impaired (Figure 3A). In cells infected with HIV-1 (WT) there was evidence of tetherin degradation (Figure 3C) and reduced cell surface tetherin levels (not shown). This correlated with enhanced mRNA expression of *cxcl10*, *il6*, and *ifnb* (Figure 3B). Tetherin could be precipitated with anti-pY antibody from CD4+ T cells infected with HIV-1ΔVpu (Figure 3C), indicating that physiological viral restriction induces its phosphorylation. Furthermore, supernatants derived from these cells were used to treat human U87MG glioblastoma cells. U87MG displays multiple blocks to HIV replication after exposure to type 1 interferon (IFN) (Goujon et al., 2013). The cells were then challenged with VSV-G pseudotyped HIV-1 to measure one-round viral replication. Consistent with *ifnb* upregulation, treatment of U87MG cells with supernatants from T cells infected with the ΔVpu, but not WT, virus inhibited one-round HIV-1 replication by 8-fold (Figure 3D). Thus, not only does tetherin restriction of viral release promote proinflammatory responses in primary CD4+ T cells, consistent with tetherin phosphorylation, but these responses are sufficient to induce a robust antiviral state in surrounding cells.

Tetherin Signaling and Phosphorylation Are Sensitive to Inhibitors of Src-Family Kinases and Dependent on Syk

In order to show that tetherin phosphorylation is essential for virus-induced signaling, we first treated tetherin-transfected 293 cells (Figure S3A), or 293/tetherin-producing virus (Figure 4A) with a broad-spectrum tyrosine kinase inhibitor Genistein. In both cases, tetherin-dependent induction of the NF-κB reporter gene was inhibited in a dose-dependent manner. In parallel experiments, induction of an NF-κB signal through the overexpression of mitochondrial antiviral signaling protein (MAVS) was unaffected (Figure S3A). Since the YDYCRV motif contains a putative SH2-binding domain, we then assessed whether tetherin-mediated signaling was sensitive to PP2, an inhibitor of Src-family kinases that phosphorylate tyrosines in these

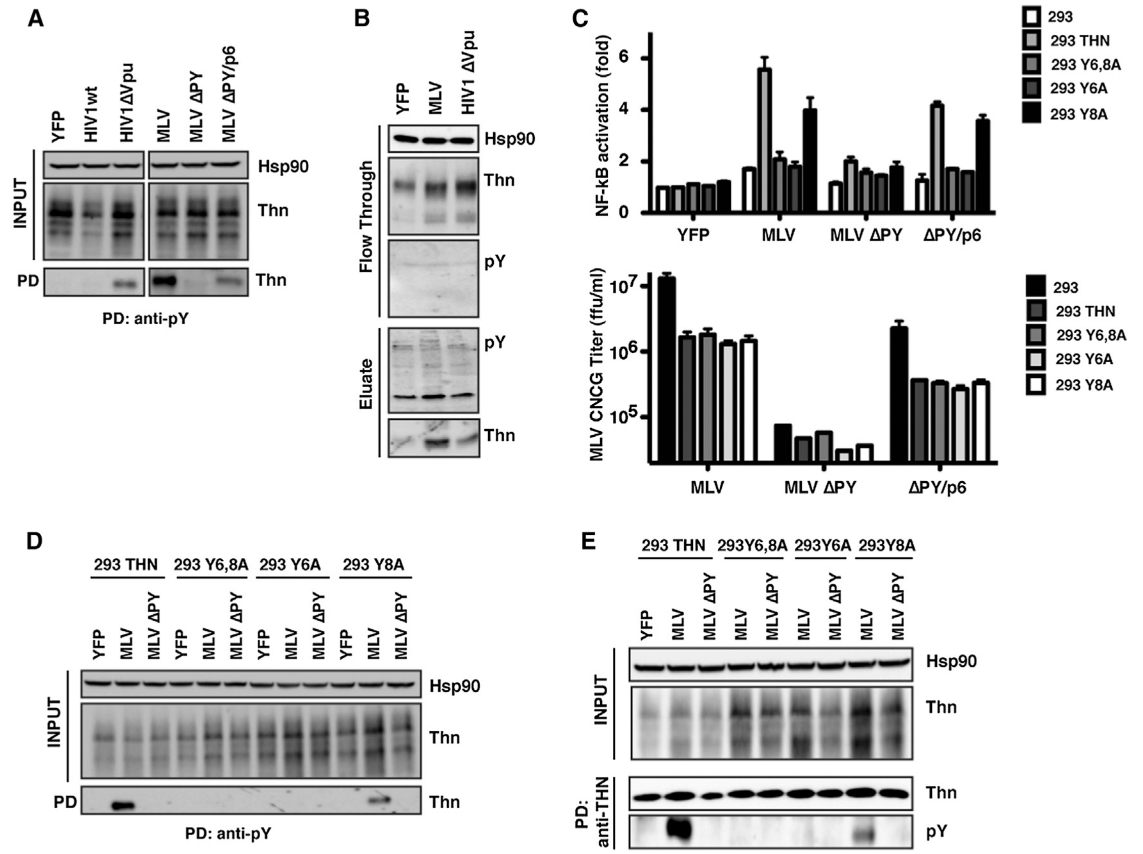

Figure 2. Retroviral Particle Retention Induces Tyrosine Phosphorylation of Human Tetherin

(A) Anti-pY immunoprecipitation of cell lysates from 293/tetherin cells transfected with wild-type and ΔVpu HIV-1 proviruses or MLV provirus and derivatives (MLVΔPY and MLVΔPY/p6). Immunoprecipitates were deglycosylated, and tetherin was detected by western blot.

(B) Phosphorylated proteins were separated from lysates of 293/tetherin cells transfected with YFP control, ΔVpu HIV-1, or MLV proviruses using a Phosphoprotein Purification Column. Tetherin was detected by western blot of the flow-through or eluted fractions.

(C) 293/tetherin, single tyrosine (Y6A, Y8A), or double-tyrosine mutants (Y6,8A) assayed for the ability to induce NF-κB-luc reporter activation upon transfection with MLV, MLVΔPY, and MLVΔPY/p6 (top). MLV infectivity was determined by titration of supernatants on 293T cells and analyzed by flow cytometry for GFP expression 48 hr later (bottom). Error bars are ± SEM of three independent experiments.

(D and E) The same cells were transfected with MLV provirus or late domain mutant MLV ΔPY, and 24 hr later cells were lysed for immunoprecipitation with anti-pY (D) or anti-tetherin (E) antibodies. Precipitates were deglycosylated and analyzed by western blot as before. See also Figure S2.

motifs. Again, we observed dose-dependent inhibition of tetherin-dependent reporter gene activity from both overexpression or cotransfection of MLV or HIV-1ΔVpu into 293/tetherin cells (Figures S3B and 4B), while noting minimal effect on both virion retention and viral assembly (Figures S3C and S3D). Importantly, both Genistein (Figure S3E) and PP2 treatment also blocked the ability to precipitate tetherin with anti-pY antibodies (Figures 4C and S3F), indicating that tetherin phosphorylation is catalyzed by Src-family kinases.

The similarities between the requirements for tetherin signaling, and the hemITAMs found in dimeric C-type lectins (Fuller et al., 2007; Sancho et al., 2009; Suzuki-Inoue et al., 2006), prompted us to test whether this was also dependent on Syk kinase binding to the tetherin phosphotyrosine motif. We first depleted Syk using two siRNAs and found that both significantly blocked NF-κB reporter gene activity from both overexpressing tetherin and virion budding (Figures S3G and 4D). Again, while neither oligonucleotide had any effect on virion release or Vpu function (Figure S3H), Syk depletion specifically blocked pull-down of phospho-tetherin (Figure 4E), suggesting it either phosphorylates tetherin directly or stabilizes the phosphorylated form. Tetherin was then immunoprecipitated from transiently transfected 293 cells (Figure S3I) or cells expressing tyrosine mutant tetherins producing MLV VLPs to determine if Syk associates with it (Figure 4F). In both cases Syk and its active phosphorylated form (pSyk) could be detected in the presence of WT tetherin, but not those bearing a Y6 mutation. Interestingly, mutation of Y8 alone did not abolish Syk recruitment, but it reduced the total amount detectable in the IP, consistent with an intermediate signaling phenotype. We and others have previously found that the E3 ligases TRAF2 and TRAF6 and their target kinase TAK1 associate with tetherin dependent on the tyrosine motif (Galão et al., 2012; Tokarev et al., 2013). Recovery of phosphorylated proteins from 293/tetherin and 293/tetherin Y6,8A cells producing MLV VLPs increased the yield of Syk, TRAF2, TRAF6, and TAK1, but not TRAF3, consistent with the

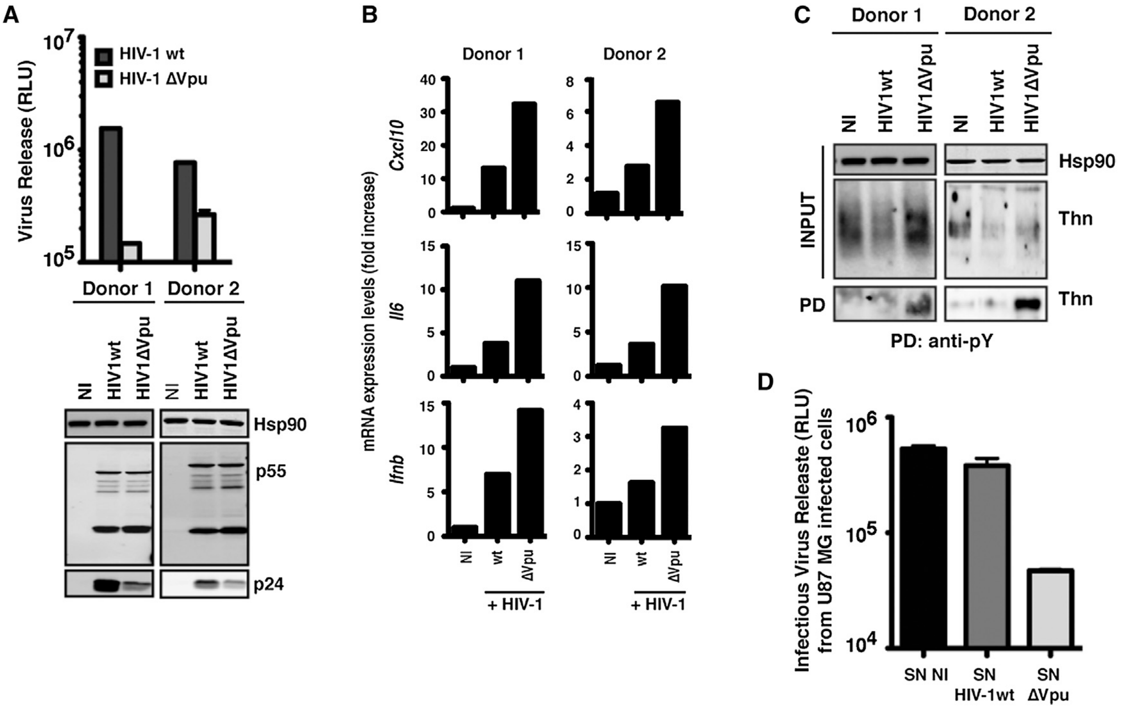

Figure 3. Restriction of Tetherin-Sensitive HIV-1 Mutant Release from CD4+ T Cells Induces Tetherin Phosphorylation

(A) Purified CD4+ T cells from two donors were infected with HIV-1 wild-type or ΔVpu virus at an moi of 3. At 48 hr later, infectious virus release was determined by infection of HeLa-TZMbl reporter cell lines and western blot of cell lysates and supernatants for HIV-1 p24-CA.

(B) Total RNA from the cells infected in (A) was analyzed for *cxcl10*, *il6*, and *ifnb* mRNA levels relative to *gapdh* by qRT-PCR.

(C) Parallel CD4+ T cell cultures were infected as in (A). Cell lysates from these cultures were immunoprecipitated with an anti-pY antibody, deglycosylated, and tetherin analyzed by western blot.

(D) U87MG cells were treated overnight with supernatants from CD4+ T cells infected as in (A) and then challenged with VSV-G pseudotyped HIV-1 WT virus (moi of 0.5) in a one-round viral replication assay. Infectious virus release was determined 48 hr later as in (A).

assembly of such a signaling complex (Figure 4G). Under the same conditions, knockdown of Syk abolished the recovery of all of these factors (Figure 4H), indicating that Syk recruitment and tetherin phosphorylation are essential for the assembly of the complex.

To confirm a role for Src family kinases and Syk in primary human cells, we then assessed the sensitivity of tetherin phosphorylation to pharmacological inhibition in infected CD4+ T cells. Under the conditions used in Figure 3, treatment with the Src inhibitor PP2 or the Syk inhibitor Piceatannol abolished tetherin phosphorylation and cytokine mRNA upregulation and their antiviral effects in cells infected with Vpu-defective HIV-1 without an effect on viral release (Figures 4I, 4J, and S3J-S3L). T cells express a highly related kinase to Syk, Zap70, which has an essential role in T cell activation downstream of ITAM motifs in subunits of the T cell receptor complex (Chu et al., 1998). In addition to higher levels of phosphorylated Syk in HIV-1ΔVpu-infected cells, we also found evidence of phospho-Zap70. Since Piceatannol inhibits both kinases, we also used a Syk-selective inhibitor, BAY61-3606. While this treatment effectively blocked the appearance of phospho-Syk, BAY61-3606 markedly reduced, but did not abolish, tetherin phosphorylation (Figure 4I). These data therefore indicate that while Syk likely represents the major effector of tetherin-mediated signal transduction in CD4+ T cells, there may also be scope for some redundancy with Zap70.

Species Specificity of Tetherin Signaling Reflects Efficiencies of Tyrosine Phosphorylation and Syk Activation

The species specificity of tetherin signaling maps to its CT, as well as a 2 amino acid insertion in the TM domain present in humans and great apes (Figure S4A) (Galão et al., 2012). The former culminates in the enhanced activity of human tetherin to activate NF-κB, while the latter suggests a conformational change in the orientation of the CT between the tetherins of hominoids and other primates. We therefore examined whether different species of tetherins could be precipitated with anti-pY antibodies upon overexpression. Consistent with their signaling capabilities (Figure 5A), only the human and chimpanzee orthologs could be precipitated in such a manner, with a trace of gorilla tetherin detectable (Figure 5B). The species-specific changes that we previously showed to impair tetherin signaling (Galão et al., 2012) (Figure S4A), as well as the Ser and Thr residues at positions 3 and 4 (Figure S4B), all impacted the efficiency with which tetherin could be precipitated from transiently transfected cells with anti-pY antibodies (Figure S4C). The direct immunoprecipitation of primate tetherins revealed that the nonsignaling tetherins reacted weakly with pY antibodies, suggesting that they can be phosphorylated to some extent (Figure 5C). However, while some Syk coprecipitated with other primate tetherins, only phosphorylated Syk could be

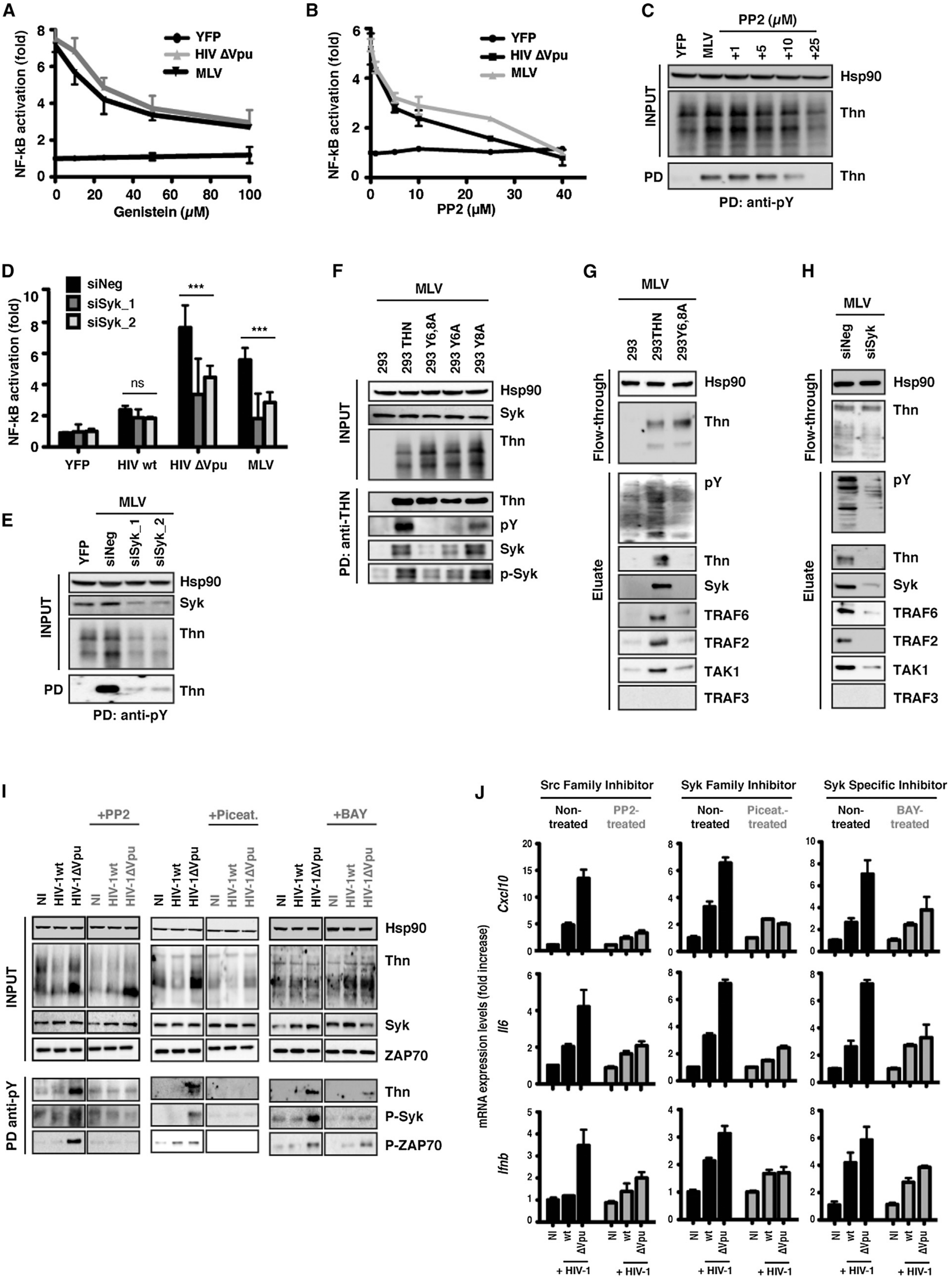

(legend on next page)

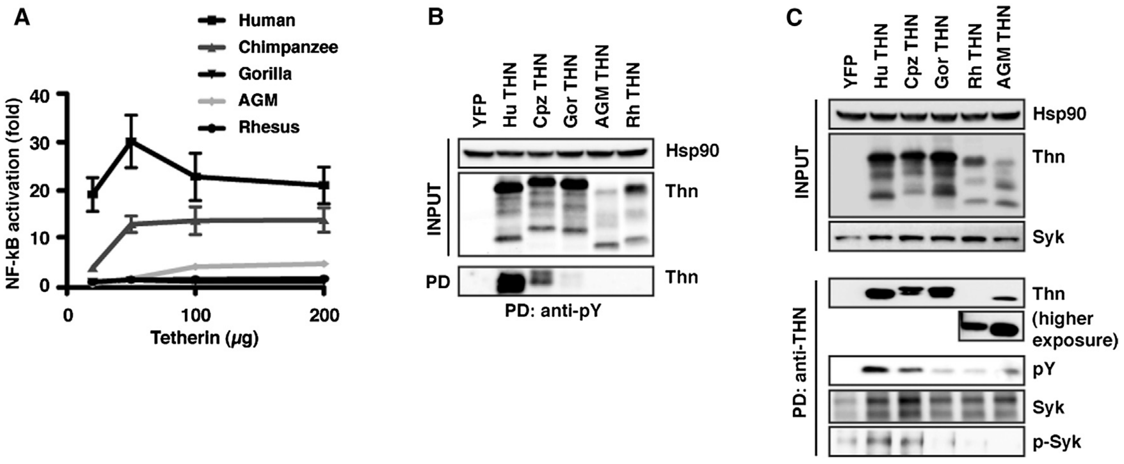

Figure 5. Species Specificity of Tetherin's Signaling Activity Is Linked to Syk Recruitment and Tyrosine Phosphorylation

(A) NF-κB-luc reporter activation in 293 cells transfected with increasing amounts of human, chimpanzee, gorilla, AGM, and rhesus tetherins. Alignment of cytoplasmic tails of primate tetherins is presented in Figure S4A. Error bars represent ± SEM of three experiments.

(B) Anti-pY immunoprecipitation of lysates from the 50 ng input of (A) deglycosylated and western blotted for tetherin.

(C) As in (B), but cell lysates were precipitated with anti-tetherin antibodies, and precipitates were analyzed for phosphorylated tetherin (anti-pY), Syk, or pSyk. See also Figure S4.

detected in association with the human and chimpanzee proteins (Figure 5C).

Tetherin-Mediated Virion Retention and Signal Transduction Are Coupled via the Actin Cytoskeleton and RICH2

Our previous observations and those herein provide evidence for the dual nature of the YDYCRV in tetherin, depending on its presence in long tetherin homodimers. In particular, our previous data indicated that blockade of tethered virion endocytosis potentiates signaling, suggesting that the clustering of tetherin dimers at the plasma membrant (PM) in assembling virions is the primary trigger (Galão et al., 2012). Interestingly, there is evidence that hemITAM function in C-type lectins requires the underlying actin cytoskeleton (Pollitt et al., 2010). Since tetherin has been previously associated with the cortical actin network (Rollason et al., 2009), we tested whether tetherin signaling upon virion retention was sensitive to actin depolymerization. Treatment of 293/tetherin cells transfected with Vpu-defective HIV-1 or MLV VLPs with cytochalasin D completely abolished the activation of the NF-κB reporter construct without affecting this response induced by MAVS overexpression (Figure 6A). By contrast, neither viral infectivity, tetherin surface expression, nor tetherin-mediated restriction of viral release were affected by this treatment (Figures 6B and S5A). Furthermore, in parallel experiments, cytochalasin D also blocked the ability to precipitate phosphorylated tetherin in MLV-transfected cells and prevented coimmunoprecipitation of phosphorylated Syk (Figure 6C). Mature tetherin localizes to detergent-resistant microdomains (DRMs) by virtue of its GPI anchor (Kupzig et al., 2003). Neither MLV proviral expression nor cytochalasin D treatment had any effect on the partitioning of tetherin into DRMs isolated on OptiPrep gradients (fraction 3, Figure S5B). Interestingly, phospho-tetherin and Syk could be immunoprecipitated only from the DRM fraction in the presence of MLV VLP assembly (Figure S5C), suggesting that tetherin-mediated virion retention is sensed through the actin cytoskeleton and is dependent on its spatial distribution at the PM.

Figure 4. Tetherin Phosphorylation Is Sensitive to Inhibitors of Src-Family Kinases and Requires the Recruitment of Syk

(A and B) Fold activation of NF-κB-luc reporter in 293/tetherin cells transfected with HIV-1 ΔVpu or MLV proviruses in the presence of increasing amounts of the inhibitors Genistein (A) or PP2 (B). NF-κB fold changes relative to cells transfected with control YFP and not treated with inhibitors.

(C) Immunoprecipitation of cell lysates from (B) with an anti-pY antibody. Precipitates from 293/tetherin cells transfected with MLV provirus were deglycosylated and analyzed as previously described.

(D) 293/tetherin cells were transfected twice over 72 hr with siRNAs directed against Syk (siSyk_1 and _2) or a nontargeting control (siNeg). 293 tetherin cells were transfected with the NF-κB reporter plasmid and the indicated viral plasmid. Fold increases in luciferase expression are plotted relative to YFP control. All error bars represent ± SEM of three experiments. *p > 0.05, **p > 0.01, and ***p > 0.001 as determined by paired two-tailed t test.

(E) Western blots of anti-pY immunoprecipitates from (D).

(F) Lysates of 293 stably expressing wild-type tetherin or tyrosine mutants transfected with MLV provirus were immunoprecipitated with anti-tetherin antibody. Pull-downs were analyzed by western blot for tetherin, pY, Syk, and its active phosphorylated form (pSyk).

(G and H) Phosphorylated proteins were separated from MLV-transfected lysates of 293/tetherin and 293/tetherin-Y6,8A (G) or 293/tetherin cells depleted of Syk by siRNA (H),using a Phosphoprotein Purification Column. Eluate fractions were analyzed by western blot for the indicated protein.

(I) Purified CD4+ T cells were infected with HIV-1 WT or ΔVpu viruses (moi of 3) in the presence or absence of the inhibitors PP2 (20 μM), Piceatannol (10 μM), or BAY61-3606 (2.5 μM). At 48 hr after infection, cell lysates were immunoprecipitated with an anti-pY antibody, and pull-downs were deglycosylated and analyzed by western blot for tetherin, pSyk, and pZAP70.

(J) *Cxcl10*, *il6*, and *ifnb* mRNA levels in parallel cultures infected and treated as in (I). Data are represented as ± SEM. See also Figure S3.

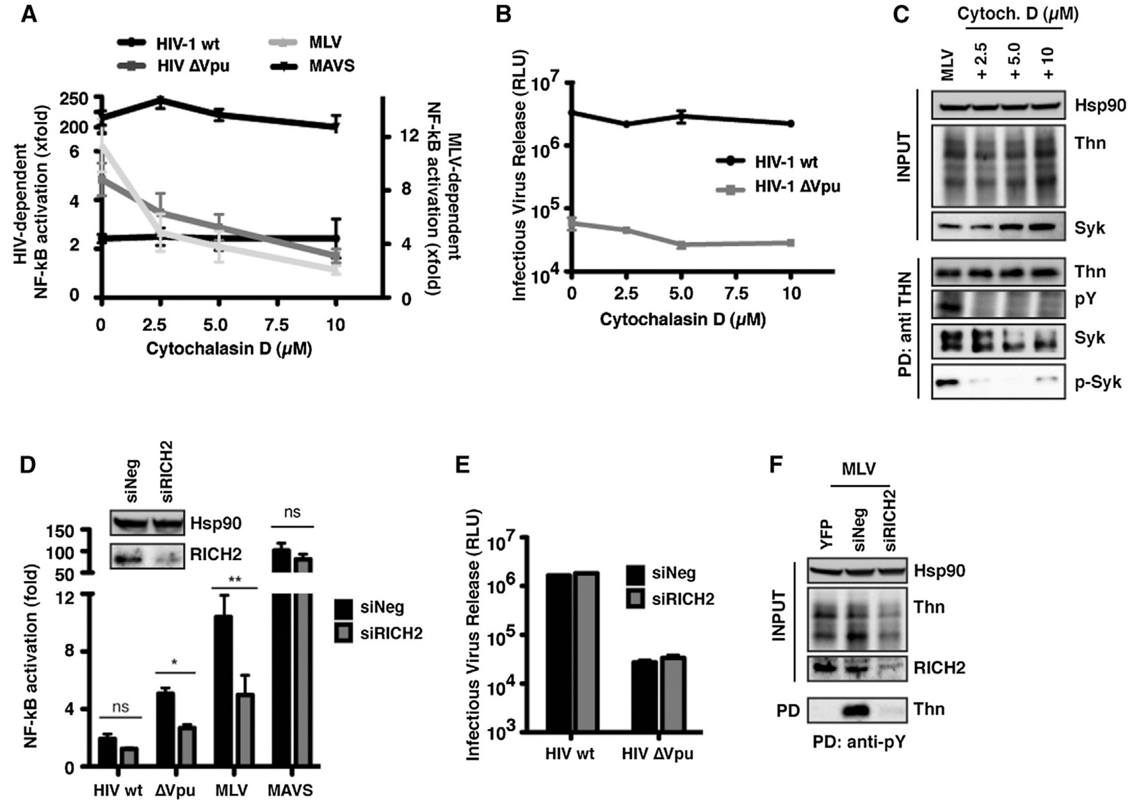

Figure 6. Tetherin-Mediated Signaling Is Impaired by Cytochalasin D Treatment and RICH2 Depletion

(A) Fold activation of NF-κB-luc reporter in 293/tetherin cells transfected with MLV provirus, wild-type, ΔVpu HIV-1 proviruses, or MAVS and treated for 6–8 hr with increasing amounts of the actin polymerization inhibitor cytochalasin D.

(B) HIV-1 infectious viral particle release from supernatants in (A) was determined on HeLa-TZM cells.

(C) 293/tetherin cells were transfected with MLV provirus in the presence of increasing amounts of cytochalasin D inhibitor. Lysates were immunoprecipitated with an anti-tetherin antibody and analyzed by western blot for anti-pY, Syk, and pSyk.

(D) NF-κB reporter activation in RICH2 siRNA-depleted 293/tetherin cells transfected with wild-type HIV-1, ΔVpu HIV-1, or MLV proviruses. Knockdown of RICH2 was confirmed by western blot (inset). Error bars represent ± SEM of three experiments. *p > 0.05, **p > 0.01, and ***p > 0.001 as determined by paired two-tailed t test.

(E and F) Infectious HIV release (E) and precipitation of phospho-tetherin by anti pY from MLV transfected cells (F) were analyzed as in previous figures. See also Figure S5.

The BAR-RacGAP protein RICH2 (ARHGAP44) has been identified to link tetherin to the cortical actin network in polarized cells (Rollason et al., 2009). We hypothesized that RICH2 might play a role in the initiation of tetherin-mediated signal transduction. Depletion of RICH2 in 293/tetherin cells by RNAi specifically blocked the ability of both HIV-1ΔVpu and MLV to induce NF-κB-luciferase (NF-κB-luc) reporter gene activity (Figure 6D) but had no effect on the release of either HIV-1ΔVpu (Figure 6E) or MLV (not shown), indicating that it plays no direct role in tetherin's physical antiviral function but is required for the initiation of signaling. In keeping with this, RICH2 RNAi blocked detection of phosphorylated tetherin under these conditions (Figure 6F).

We then examined RICH2 interaction with tetherin. An N-terminally hemagglutinin (HA)-tagged human RICH2 coprecipitated with human, ape, and rhesus macaque, but not African green monkey (AGM), tetherins (Figure 7A), indicating the determinants of interaction are at least partially conserved and do not correlate with signaling capacity. The juxtamembrane region of the CT of tetherin is more conserved in primates (Figure S4A), and most mutations in this region of human tetherin have little effect on signaling. However, rhesus and AGM tetherins differ at two positions (21 and 24). Interestingly, a recent study has documented a rare polymorphism in the human tetherin CT, R19H (Sauter et al., 2013). The R at the equivalent position (24) is shared in rhesus, but not AGM, tetherin. In the context of the human protein, the R19H polymorphism reduced efficiency of NF-κB activation without affecting antiviral activity (Sauter et al., 2013). In agreement with these data, we found that NF-κB activation by tetherin-R19H was reduced 5- to 6-fold compared to the wild-type protein when overexpressed by transient transfection (Figure S6A). When the protein was stably expressed in 293 cells, HIVΔVpu virion retention by tetherin R19H failed to mediate NF-κB activation (Figures 7B and S6B). Furthermore, no evidence of tetherin phosphorylation was observed in 293/tetherin-R19H cells producing MLV VLPs (Figure 7C). Interestingly, tetherin R19H displayed a marked reduction in the ability to coprecipitate HA-tagged RICH2 (Figure 7D), suggesting that a defect in this interaction underlies the signaling impairment

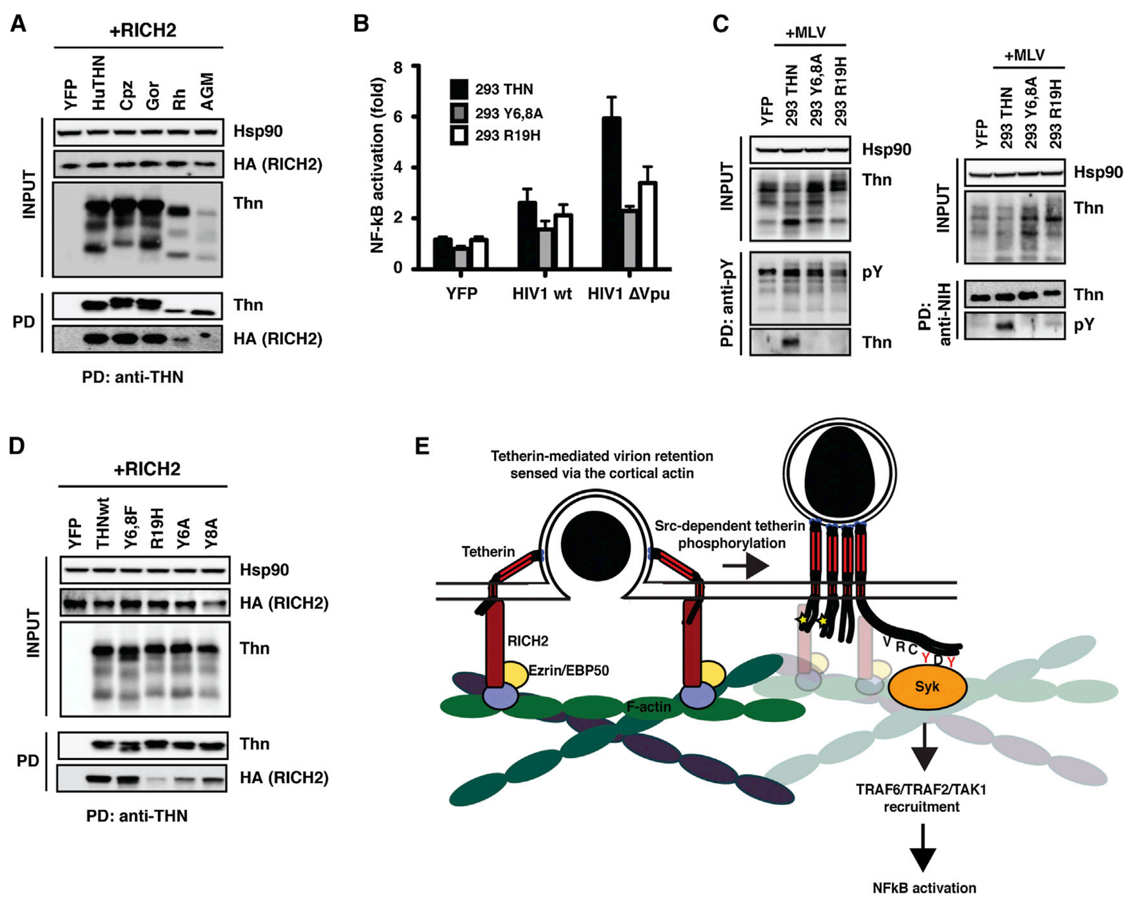

Figure 7. RICH2/Tetherin Interactions Are Required for Virion-Induced Signaling

(A) 293 cells were cotransfected with an N-terminally HA-tagged human RICH2 and tetherin from humans, chimpanzee, gorilla, rhesus, or AGM. Lysates were immunoprecipitated with an anti-tetherin antibody, and precipitates were blotted for HA and tetherin.

(B) Fold activation of a NF-κB-luc reporter in 293 cells expressing tetherin, or the mutants Y6,8A and R19H, transfected with HIV-1 WT or ΔVpu proviruses relative to YFP control. Error bars represent ± SEM of three experiments.

(C) The above stable cells lines were transfected with MLV provirus and lysed 24 hr later for immunoprecipitation with a anti-pY (left) or anti-tetherin (right) antibodies. Precipitates were deglycosylated and analyzed by western blot as before.

(D) 293 cells were cotransfected with HA-RICH2 and wild-type tetherin or the mutants Y6,8A, Y6A, Y8A, or R19H. Lysates were immunoprecipitated with an anti-tetherin antibody and analyzed as in (A).

(E) Model for Syk-dependent tetherin-mediated signaling as discussed in the Discussion. See also Figure S6.

of this mutant. Unlike the dual-tyrosine mutant, the R19H polymorphism also displayed only weak dominant interfering activity (Figure S6C), in agreement with the previous study (Sauter et al., 2013). Together, these data indicate that RICH2/tetherin interactions are important to trigger the cytoskeletal-dependent activation of tetherin signaling downstream of virion retention. Furthermore, this interaction is defined in part by a juxtamembrane determinant in the tetherin CT that defines a rare signaling polymorphism in humans.

DISCUSSION

In this study we have probed the mechanism by which restriction of viral release by human tetherin triggers proinflammatory signaling (Figure 7E). We find results consistent with the YDYCRV motif in tetherin acting like hemITAMs found in several dimeric C-type lectins. Retroviral assembly and release is "sensed" through tetherin's link to the cortical actin network via RICH2, which leads to tyrosine phosphorylation of tetherin by Src-family kinases and recruitment of Syk. This in turn promotes NF-κB activation dependent on TRAFs 2 and 6 and TAK1, as we and others have previously shown (Galão et al., 2012; Tokarev et al., 2013). Together, these data provide a plausible model for how an infected cell can detect the illicit budding of enveloped virions at plasma membrane through the antiviral activity of tetherin. Furthermore, the species specificity of this mechanism suggests that changes in the tetherin CT and its orientation during hominid evolution permitted the dual-tyrosine trafficking motif to be phosphorylated and act as an SH2-binding site for Syk.

This mechanism of hemITAM signaling in tetherin is very similar to that described for the C-type lectins CLEC2, Dectin-1, and DNGR-1 (Fuller et al., 2007; Sancho et al., 2009; Suzuki-Inoue et al., 2006). In all cases, these are type II membrane

proteins with short CTs that either are constitutively dimeric or dimerize upon ligand binding. CLEC2 binding of its ligand, rhodocytin, leads to its translocation to lipid rafts and phosphorylation by Src in an actin-dependent manner (Pollitt et al., 2010). This recruits Syk to the phosphorylated YXXL SH2-binding site (Séverin et al., 2011). Tetherin constitutively resides in cholesterol-rich microdomains by virtue of its GPI anchor (an essential attribute for signaling) (Kupzig et al., 2003). We propose that surface clustering of tetherin by virus budding is sensed similarly through the actin cytoskeleton. In contrast to other hemITAMs, tetherin bears an unusual dual-tyrosine motif, of which Y6 is essential. Surprisingly, mutation of Y8, which is in the context of a canonical SH2-binding domain, affects the efficiency of signaling and Syk recruitment but does not abolish it. Furthermore, a Y8H polymorphism that maintains signaling and antiviral activity in transient assays has been detected in humans (Sauter et al., 2013). Since phosphorylation of either tyrosine has been detected in phosphoproteome studies (St-Germain et al., 2009), a potential explanation might be that initial Y6 phosphorylation by Src-family kinases (or Syk itself) leads to recruitment of Syk, which then phosphorylates Y8 to stabilize the interaction. How the TRAF2/TRAF6/TAK1 complex is recruited remains to be determined. If tetherin signaling is similar to other hemITAMs, then the route to NF-κB may be through phospholipase C, PKC, and the CARD9/Bcl10/MALT1 complex (Strasser et al., 2012). Whether tetherin-mediated restriction modulates other pattern recognition pathways through Syk is another interesting question. Dectin-1 ligation leads to inflammasome activation through Syk in combination with Toll-like receptor 2 (TLR2) (Hise et al., 2009), and we have previously shown that tetherin-dependent type 1 IFN production in T cells may also require a TLR/TRIF pathway (Galão et al., 2012). Furthermore, tetherin has been proposed as a cofactor for the leukocyte inhibitory receptor ILT-7, which suppresses TLR responses in plasmacytoid dendritic cells (pDCs) in combination with FcεRIγ (Cao et al., 2009). While these observations are controversial (Tavano et al., 2013), they highlight scope for tetherin signaling to impact upon the regulation of other innate immune responses.

The implication of RICH2 in the mechanism of tetherin signaling is intriguing. RICH2, a BAR-domain containing RacGAP was identified several years ago as linking tetherin to the subapical cortical actin in polarized epithelial cells via ezrin and EBP50 (Rollason et al., 2009). This interaction was required for the maintenance of the cortical actin network, and its disruption led to the activation of the GTPase Rac. While such a phenotype may not be consistent with the apparently normal development of tetherin knockout mice (Liberatore and Bieniasz, 2011), these data suggest that actin dynamics may play a role in tetherin's activity in vivo. Our data indicate that RICH2 and the cortical actin are not required for restriction of virion release, consistent with tetherin's proposed mechanism of virion entrapment (Venkatesh and Bieniasz, 2013). However, coupling tetherin to actin via RICH2 provides a plausible explanation for our previous observations that tetherin clustering by crosslinking or virion budding is the primary trigger of signal transduction (Galão et al., 2012) and suggests a mechanism through which Src-family kinases can be activated. RICH2 is unlikely to simply be required for tetherin-dependent signaling. Human RICH2 was initially isolated from cell lysates using the rat tetherin CT as bait, suggesting cross-species conservation of such an interaction (Rollason et al., 2009). Data herein indicate that human RICH2 interacts with a subset of signaling and nonsignaling primate tetherins. Although the molecular determinants are yet to be fully described, RICH2 interactions with human tetherin are affected by a rare natural polymorphism previously found to be defective for signal transduction (R19H). The equivalent position varies between rhesus and AGM tetherins, which differ in human RICH2 interactions despite both being unable to signal in human cells. The RICH2-mediated linkage to actin may also be required for the internalization of tethered virions for endosomal degradation. This internalization would also likely require actin dynamics, and hence a coupling to Rho-family GTPases, whose role in both tetherin signaling and virion internalization remains to be determined. Interestingly, a rare synonymous polymorphism in RICH2 adjacent to the splice acceptor in exon 11 has been associated with slow progression to AIDS (Le Clerc et al., 2011), raising a possibility that the R19H tetherin polymorphism or differential expression of splice variants of RICH2 may affect tetherin's function in vivo.

Our data have shed further light on the species specificity of tetherin signaling, particularly as the virtual invariance of the Syk and RICH2 sequences in primates argues against either determining the lack of signaling activity in monkey tetherins. Changes in the tetherin CT and a 2 amino acid TM insertion that occurred during hominoid evolution have generated a signaling capacity coupled to its antiviral action (Galão et al., 2012). This correlates with tetherin phosphorylation and Syk activation. We suggest that a potential conformational change in the CT produced by the lengthening of TM domain changed the position of the tyrosine residues such that Y6 was amenable to phosphorylation. Importantly this was accompanied by KM-to-RV changes adjacent to Y8 as well as residues upstream of Y6 that facilitated interaction with Syk to create a noncanonical hemITAM. Finally, the 5 amino acid indel that occurred after hominids and chimpanzees diverged (Sauter et al., 2011) enhanced this signaling capacity (Galão et al., 2012). Since some of these differences encompass residues that have been identified as modulating sensitivity to lentiviral Nefs (the indel) and Vpus (the TM insertion) (reviewed in Neil, 2013), we speculate that at least some of these changes may have resulted from pathogen counteraction of tetherin's antiviral activity. However, the dual-tyrosine motif is present in the long isoforms of the majority of mammalian tetherins, irrespective of signaling activity. This motif is required for tetherin recycling via the *trans*-Golgi network (TGN) and has been shown to bind to both AP1 and AP2 (Rollason et al., 2007). By contrast, tetherin-retained virions are internalized and targeted to late endosomes (Neil et al., 2008), suggesting that the act of viral restriction modulates tetherin trafficking. Since most mammalian tetherins do not signal, why are the two isoforms (with and without the dual tyrosines; Cocka and Bates, 2012) maintained? A possibility is that a dual motif in both long-tetherin homodimers and mixed heterodimers acts as the switch that targets virions for degradation, whereas stabilization of the short isoform at the surface could enhance opsonization. Indeed, endosomal virion localization is reduced when the tyrosines are mutated (Galão et al., 2012). In addition to virion degradation, it may afford certain cell types the ability to turn viral antigens over for presentation or expose virion components to

endosomal pattern recognition receptors (PRRs) (Galão et al., 2012). Coupling the phosphorylation to downstream internalization may also be important for damping down tetherin signaling by targeting activated molecules for endosomal degradation. Such a preexisting function of the tyrosine motif is an attractive starting point from which to evolve a further function, namely proinflammatory signaling coupled to tetherin's antiviral activity. It also raises questions of whether tetherin function is compromised in mammals that lack expression of the long isoform entirely, as suggested by the recent studies showing that expression of both isoforms qualitatively influences innate and adaptive immune responses to MLV infection in mice (Li et al., 2014).

The long isoform of human tetherin is more efficiently targeted by M group HIV-1 Vpu because, in contrast to the short form, it can induce its degradation (Cocka and Bates, 2012; Weinelt and Neil, 2014). This differential sensitivity and targeted degradation is not observed in lentiviral countermeasures from viruses that infect primate species whose tetherins lack NF-κB activation (Weinelt and Neil, 2014). Furthermore, the acquisition of long tetherin isoform degradation in only HIV-1 group M tempts speculation that tetherin's signaling capacity had a major effect on the adaptation of that zoonotic infection to become the HIV/AIDS pandemic.

EXPERIMENTAL PROCEDURES

Plasmids and Cells

Human and primate tetherin constructs, or mutants thereof, were made by standard techniques in pCR3.1 (Invitrogen) or pLHCX (Clontech). Tetherin-expressing 293 cells were generated by retroviral vector transduction of pLHCX constructs followed by hygromycin selection. HIV-1 and MLV proviral/vector plasmids and firefly luciferase NF-κB and *Renilla* control reporter plasmids were described previously (Galão et al., 2012) and detailed in the Supplemental Experimental Procedures. A codon-optimized human RICH2 was synthesized (MWG) and cloned into pCR3.1 with an N-terminal HA tag. CD4+ T cells were isolated from healthy donors using an Untouched CD4+ T cell Dynabead kit (Life Technologies), activated for 48 hr prior to infection with anti-CD3/CD28 dynabeads (Life Technologies), and cultured in 30 U/ml human IL-2.

Reporter Gene Assays

Reporter gene assays in 293 cells transiently transfected with tetherin expression vectors were performed as described previously (Galão et al., 2012) using a Promega Dual luciferase kit. For viral-mediated reporter gene assays, 293 cells or 293 cells stably expressing tetherin were transfected with 10 ng of 3xkB-pCONA-FLuc and of pCMV-RLuc in combination with 600 ng of HIV or MLV proviruses. For experiments with MLV or derivatives, 200 ng of the MLV GFP-encoding vector, pCNCG, and 100 ng of pVSV-G were cotransfected to allow infectious release to be measured (see below).

For reporter gene assays in the presence of inhibitors, cells were transfected as described above. At 4 hr posttransfection, cells were treated for 6–8 hr with increasing amounts of the inhibitors Genistein (Source Bioscience), PP2 (Sigma), or cytochalasin D (Sigma), after which fresh medium was added. Tetherin expression on the cell surface was determined by flow cytometry using anti-human BST2-PE (eBiosciences). The irreversible Syk inhibitors Piceatannol (Invivogen) and BAY61-3606 (Sigma) were used to treat infected primary T cells for 8 hr, and cells were harvested 24 hr later.

For RNAi knockdown of Syk and RICH2, 2×10^5 cells were seeded per well of a 12-well plate and transfected 6 hr later with 100 pmol of siRNA in complex with Dharmafect-1 (Dharmacon). At 48 hr later, cells were reseeded and cotransfected with a second dose of siRNA alongside reporter constructs as per the transient reporter gene assays. The following siRNAs were used: Hs_SYK_6 and Hs_SYK_8 (QIAGEN: SI02223151 and SI02655135), siNegative control siRNA duplex (1027310), and Thermo Scientific ON-TARGETplus SMARTpool for human ARHGAP44 (RICH2) (L-009238-01). Protein knockdowns were verified by western blot with antibodies detailed in the Supplemental Experimental Procedures.

Virus Stocks, Primary Cell Infections, and Release Assays

HIV-1 NL4.3 wild-type and ΔVpu viral stocks were produced in 293T cells by transient transfection. Viral stocks were treated for 2 hr with 10 U/ml DNase-I (Roche) and 10 μM $MgCl_2$ and then concentrated by ultracentrifugation through a 20% sucrose/PBS cushion (30,000 rpm on a SuperSpin 360 rotor for 90 min) and resuspended in RPMI. Endpoint titers were determined on the HeLa-TZM reporter cell line as described previously (Galão et al., 2012).

For viral release assays in primary HIV target cells, 5×10^5 activated CD4+ T cells were infected at an moi of 3 to ensure >90% p24+ cells 48 hr postinfection. HIV-1 infection of T cells was monitored by flow cytometry using intracellular anti-p24-PE staining (Beckman Coulter). The infectivity of viral supernatants was determined on HeLa TZM cells, while HIV-1 particle release was determined by western blot as previously described (Galão et al., 2012). Cells were also harvested for immunoprecipitation studies, and total RNA was isolated, reverse transcribed, and resultant cDNA was used for quantitative RT-PCR to determine relative mRNA expression levels of *cxcl10*, *il6*, *and ifnb* relative to *gapdh* by qRT-PCR using TaqMan assay (ABI) primer probes. MLV vector titers were determined by transducing 293T cells and enumerating the GFP-positive cells by fluorescence-activated cell sorting 48 hr later.

Antiviral Activities of Conditioned Media from Infected CD4+ T Cells

To assess the antiviral activity of supernatants from HIV-1-infected CD4+ T cells, 10^5 U87MG glioblastoma cells, which are devoid of HIV receptor expression and therefore cannot be infected by virus released into the T cell supernatant, were treated with 200 μl of conditioned medium for 24 hr. The cells were then challenged with VSV-G pseudotyped WT HIV-1 at an moi of 0.5. The medium was changed 6 hr later, and 48 hr postinfection, infectivity in the medium was assessed on HeLa-TZM indicator cells as a measure of one-round virus replication.

Immunoprecipitations and Purification of Phosphorylated Proteins

For transient assays, 293 cells were transfected with 500 ng of pCR3.1-YFP or pCR3.1 tetherin constructs using Lipofectamine. For viral-based assays, 10 cm dishes of 293 cells stably expressing different tetherins were transfected with 6 μg of HIV or MLV proviruses. At 24 hr posttransfection, cells were lysed with a buffer containing 50 mM Tris-HCl (pH 7.4), 150 mM NaCl, 0.1% SDS, 0.5% sodium deoxycholate, 1% NP-40, 200 μM sodium orthovanadate, and protease inhibitors (Roche). Following sonication, lysates were immunoprecipitated with either mouse anti-phosphotyrosine (clone 4G10) (Millipore) or rabbit anti-BST2 polyclonal (kindly provided by K. Strebel through the NIH ARPP; Miyagi et al., 2009) antibodies and protein G agarose beads (Life Technologies) for 3 hr. Proteins bound to the beads were then deglycosylated by an overnight treatment with PNGase F (NEB).

For the separation of phosphorylated and unphosphorylated proteins from cell lysates, a PhosphoproteinProtein Purification kit (QIAGEN) was used, following the manufacturer's instructions. Total eluates were concentrated using Nanosep Ultrafiltration Columns (QIAGEN) down to a final volume of 50 μl.

Cell lysates, IPs, and eluates were subjected to SDS-PAGE and western blots performed using antibodies against BST2, pY, and cellular factors detailed in the Supplemental Experimental Procedures. Blots were visualized by ImageQuant using anti-mouse or anti-rabbit HRP-linked antibodies (NEB) or an anti-goat HRP-linked antibody (Santa Cruz Biotechnology).

Statistical Analysis

Statistical significance was determined using paired two-tailed t tests calculated using the Prism software. Significance was ascribed to p values as follows: $*p > 0.05$, $**p > 0.01$, and $***p > 0.001$.

Ethical Approval

Approval to isolate and propagate T cells from fresh blood from healthy consenting volunteers was given by the KCL Infectious Diseases BioBank Local Research Ethics Committee (approval number SN-1/6/7/9).

SUPPLEMENTAL INFORMATION

Supplemental Information includes Supplemental Experimental Procedures and six figures and can be found with this article online at http://dx.doi.org/10.1016/j.chom.2014.08.005.

AUTHOR CONTRIBUTIONS

R.P.G., S.P., and S.J.D.N. planned the study. R.P.G. and S.P. performed the experiments. R.C. contributed new reagents. R.P.G., S.P., and S.J.D.N. analyzed the data and wrote the paper.

ACKNOWLEDGMENTS

We thank George Banting, Juan Martin-Serrano, and members of the Neil lab for support and advice. This work was funded by a European Research Council Consolidator Grant (281598) and a Wellcome Trust Senior Research Fellowship (WT098049AIA) to S.J.D.N.

Received: May 1, 2014
Revised: July 18, 2014
Accepted: August 7, 2014
Published: September 10, 2014

REFERENCES

Arias, J.F., Heyer, L.N., von Bredow, B., Weisgrau, K.L., Moldt, B., Burton, D.R., Rakasz, E.G., and Evans, D.T. (2014). Tetherin antagonism by Vpu protects HIV-infected cells from antibody-dependent cell-mediated cytotoxicity. Proc. Natl. Acad. Sci. USA *111*, 6425–6430.

Barrett, B.S., Smith, D.S., Li, S.X., Guo, K., Hasenkrug, K.J., and Santiago, M.L. (2012). A single nucleotide polymorphism in tetherin promotes retrovirus restriction in vivo. PLoS Pathog. *8*, e1002596.

Cao, W., Bover, L., Cho, M., Wen, X., Hanabuchi, S., Bao, M., Rosen, D.B., Wang, Y.H., Shaw, J.L., Du, Q., et al. (2009). Regulation of TLR7/9 responses in plasmacytoid dendritic cells by BST2 and ILT7 receptor interaction. J. Exp. Med. *206*, 1603–1614.

Chu, D.H., Morita, C.T., and Weiss, A. (1998). The Syk family of protein tyrosine kinases in T-cell activation and development. Immunol. Rev. *165*, 167–180.

Cocka, L.J., and Bates, P. (2012). Identification of alternatively translated Tetherin isoforms with differing antiviral and signaling activities. PLoS Pathog. *8*, e1002931.

Fuller, G.L., Williams, J.A., Tomlinson, M.G., Eble, J.A., Hanna, S.L., Pöhlmann, S., Suzuki-Inoue, K., Ozaki, Y., Watson, S.P., and Pearce, A.C. (2007). The C-type lectin receptors CLEC-2 and Dectin-1, but not DC-SIGN, signal via a novel YXXL-dependent signaling cascade. J. Biol. Chem. *282*, 12397–12409.

Galão, R.P., Le Tortorec, A., Pickering, S., Kueck, T., and Neil, S.J. (2012). Innate sensing of HIV-1 assembly by Tetherin induces NFκB-dependent proinflammatory responses. Cell Host Microbe *12*, 633–644.

Götz, N., Sauter, D., Usmani, S.M., Fritz, J.V., Goffinet, C., Heigele, A., Geyer, M., Bibollet-Ruche, F., Learn, G.H., Fackler, O.T., et al. (2012). Reacquisition of Nef-mediated tetherin antagonism in a single in vivo passage of HIV-1 through its original chimpanzee host. Cell Host Microbe *12*, 373–380.

Goujon, C., Schaller, T., Galão, R.P., Amie, S.M., Kim, B., Olivieri, K., Neil, S.J., and Malim, M.H. (2013). Evidence for IFNα-induced, SAMHD1-independent inhibitors of early HIV-1 infection. Retrovirology *10*, 23.

Hise, A.G., Tomalka, J., Ganesan, S., Patel, K., Hall, B.A., Brown, G.D., and Fitzgerald, K.A. (2009). An essential role for the NLRP3 inflammasome in host defense against the human fungal pathogen Candida albicans. Cell Host Microbe *5*, 487–497.

Kupzig, S., Korolchuk, V., Rollason, R., Sugden, A., Wilde, A., and Banting, G. (2003). Bst-2/HM1.24 is a raft-associated apical membrane protein with an unusual topology. Traffic *4*, 694–709.

Le Clerc, S., Coulonges, C., Delaneau, O., Van Manen, D., Herbeck, J.T., Limou, S., An, P., Martinson, J.J., Spadoni, J.L., Therwath, A., et al. (2011). Screening low-frequency SNPS from genome-wide association study reveals a new risk allele for progression to AIDS. J. Acquir. Immune Defic. Syndr. *56*, 279–284.

Li, S.X., Barrett, B.S., Heilman, K.J., Messer, R.J., Liberatore, R.A., Bieniasz, P.D., Kassiotis, G., Hasenkrug, K.J., and Santiago, M.L. (2014). Tetherin promotes the innate and adaptive cell-mediated immune response against retrovirus infection in vivo. J. Immunol. *193*, 306–316.

Liberatore, R.A., and Bieniasz, P.D. (2011). Tetherin is a key effector of the antiretroviral activity of type I interferon in vitro and in vivo. Proc. Natl. Acad. Sci. USA *108*, 18097–18101.

Martin-Serrano, J., Perez-Caballero, D., and Bieniasz, P.D. (2004). Context-dependent effects of L domains and ubiquitination on viral budding. J. Virol. *78*, 5554–5563.

Miyagi, E., Andrew, A.J., Kao, S., and Strebel, K. (2009). Vpu enhances HIV-1 virus release in the absence of Bst-2 cell surface down-modulation and intracellular depletion. Proc. Natl. Acad. Sci. USA *106*, 2868–2873.

Neil, S.J. (2013). The antiviral activities of tetherin. Curr. Top. Microbiol. Immunol. *371*, 67–104.

Neil, S.J., Eastman, S.W., Jouvenet, N., and Bieniasz, P.D. (2006). HIV-1 Vpu promotes release and prevents endocytosis of nascent retrovirus particles from the plasma membrane. PLoS Pathog. *2*, e39.

Neil, S.J., Zang, T., and Bieniasz, P.D. (2008). Tetherin inhibits retrovirus release and is antagonized by HIV-1 Vpu. Nature *451*, 425–430.

Perez-Caballero, D., Zang, T., Ebrahimi, A., McNatt, M.W., Gregory, D.A., Johnson, M.C., and Bieniasz, P.D. (2009). Tetherin inhibits HIV-1 release by directly tethering virions to cells. Cell *139*, 499–511.

Pickering, S., Hué, S., Kim, E.Y., Reddy, S., Wolinsky, S.M., and Neil, S.J. (2014). Preservation of tetherin and CD4 counter-activities in circulating Vpu alleles despite extensive sequence variation within HIV-1 infected individuals. PLoS Pathog. *10*, e1003895.

Pollitt, A.Y., Grygielska, B., Leblond, B., Désiré, L., Eble, J.A., and Watson, S.P. (2010). Phosphorylation of CLEC-2 is dependent on lipid rafts, actin polymerization, secondary mediators, and Rac. Blood *115*, 2938–2946.

Rollason, R., Korolchuk, V., Hamilton, C., Schu, P., and Banting, G. (2007). Clathrin-mediated endocytosis of a lipid-raft-associated protein is mediated through a dual tyrosine motif. J. Cell Sci. *120*, 3850–3858.

Rollason, R., Korolchuk, V., Hamilton, C., Jepson, M., and Banting, G. (2009). A CD317/tetherin-RICH2 complex plays a critical role in the organization of the subapical actin cytoskeleton in polarized epithelial cells. J. Cell Biol. *184*, 721–736.

Sancho, D., Joffre, O.P., Keller, A.M., Rogers, N.C., Martínez, D., Hernanz-Falcón, P., Rosewell, I., and Reis e Sousa, C. (2009). Identification of a dendritic cell receptor that couples sensing of necrosis to immunity. Nature *458*, 899–903.

Sauter, D., Schindler, M., Specht, A., Landford, W.N., Münch, J., Kim, K.A., Votteler, J., Schubert, U., Bibollet-Ruche, F., Keele, B.F., et al. (2009). Tetherin-driven adaptation of Vpu and Nef function and the evolution of pandemic and nonpandemic HIV-1 strains. Cell Host Microbe *6*, 409–421.

Sauter, D., Vogl, M., and Kirchhoff, F. (2011). Ancient origin of a deletion in human BST2/Tetherin that confers protection against viral zoonoses. Hum. Mutat. *32*, 1243–1245.

Sauter, D., Unterweger, D., Vogl, M., Usmani, S.M., Heigele, A., Kluge, S.F., Hermkes, E., Moll, M., Barker, E., Peeters, M., et al. (2012). Human tetherin exerts strong selection pressure on the HIV-1 group N Vpu protein. PLoS Pathog. *8*, e1003093.

Sauter, D., Hotter, D., Engelhart, S., Giehler, F., Kieser, A., Kubisch, C., and Kirchhoff, F. (2013). A rare missense variant abrogates the signaling activity of tetherin/BST-2 without affecting its effect on virus release. Retrovirology *10*, 85.

Serra-Moreno, R., Jia, B., Breed, M., Alvarez, X., and Evans, D.T. (2011). Compensatory changes in the cytoplasmic tail of gp41 confer resistance to tetherin/BST-2 in a pathogenic nef-deleted SIV. Cell Host Microbe *9*, 46–57.

Séverin, S., Pollitt, A.Y., Navarro-Nuñez, L., Nash, C.A., Mourão-Sá, D., Eble, J.A., Senis, Y.A., and Watson, S.P. (2011). Syk-dependent phosphorylation of CLEC-2: a novel mechanism of hem-immunoreceptor tyrosine-based activation motif signaling. J. Biol. Chem. *286*, 4107–4116.

St-Germain, J.R., Taylor, P., Tong, J., Jin, L.L., Nikolic, A., Stewart, I.I., Ewing, R.M., Dharsee, M., Li, Z., Trudel, S., and Moran, M.F. (2009). Multiple myeloma phosphotyrosine proteomic profile associated with FGFR3 expression, ligand activation, and drug inhibition. Proc. Natl. Acad. Sci. USA *106*, 20127–20132.

Strasser, D., Neumann, K., Bergmann, H., Marakalala, M.J., Guler, R., Rojowska, A., Hopfner, K.P., Brombacher, F., Urlaub, H., Baier, G., et al. (2012). Syk kinase-coupled C-type lectin receptors engage protein kinase C-σ to elicit Card9 adaptor-mediated innate immunity. Immunity *36*, 32–42.

Suzuki-Inoue, K., Fuller, G.L., García, A., Eble, J.A., Pöhlmann, S., Inoue, O., Gartner, T.K., Hughan, S.C., Pearce, A.C., Laing, G.D., et al. (2006). A novel Syk-dependent mechanism of platelet activation by the C-type lectin receptor CLEC-2. Blood *107*, 542–549.

Tavano, B., Galao, R.P., Graham, D.R., Neil, S.J., Aquino, V.N., Fuchs, D., and Boasso, A. (2013). Ig-like transcript 7, but not bone marrow stromal cell antigen 2 (also known as HM1.24, tetherin, or CD317), modulates plasmacytoid dendritic cell function in primary human blood leukocytes. J. Immunol. *190*, 2622–2630.

Tokarev, A., Suarez, M., Kwan, W., Fitzpatrick, K., Singh, R., and Guatelli, J. (2013). Stimulation of NF-κB activity by the HIV restriction factor BST2. J. Virol. *87*, 2046–2057.

Veillette, M., Désormeaux, A., Medjahed, H., Gharsallah, N.E., Coutu, M., Baalwa, J., Guan, Y., Lewis, G., Ferrari, G., Hahn, B.H., et al. (2014). Interaction with cellular CD4 exposes HIV-1 envelope epitopes targeted by antibody-dependent cell-mediated cytotoxicity. J. Virol. *88*, 2633–2644.

Venkatesh, S., and Bieniasz, P.D. (2013). Mechanism of HIV-1 virion entrapment by tetherin. PLoS Pathog. *9*, e1003483.

Weinelt, J., and Neil, S.J. (2014). Differential sensitivities of tetherin isoforms to counteraction by primate lentiviruses. J. Virol. *88*, 5845–5858.

Cell Host & Microbe, Volume *16*

Supplemental Information

Retroviral Retention Activates a Syk-Dependent HemITAM in Human Tetherin

Rui Pedro Galão, Suzanne Pickering, Rachel Curnock, and Stuart J.D. Neil

Supplemental Information

Supplemental Figure Inventory

Figure S1 (related to Figure 1): Transient co-expression of tetherin mutants Y6A or Y8A have a dominant-negative effect on tyrosine phosphorylation of wild-type tetherin

Figure S2 (related to Figure 2): Tetherin phosphorylation induced by retroviral assembly.

Figure S3 (related to figure 4): Treatment of cells with Tyrosine Kinase inhibitors or Syk siRNA depletion.

Figure S4 (related to Figure 5): Multiple context-dependent adaptations have resulted in the signaling capacity of human tetherin.

Figure S5 (related to Figure 6): Cytochalasin D treatment does not affect tetherin surface expression, and tyrosine phosphorylation of tetherin occurs preferentially in detergent resistant membranes.

Figure S6 (related to Figure 7): Tetherin R19H polymorphism is defective for tetherin-mediated NFkB activation but not virion retention.

Supplemental Figures

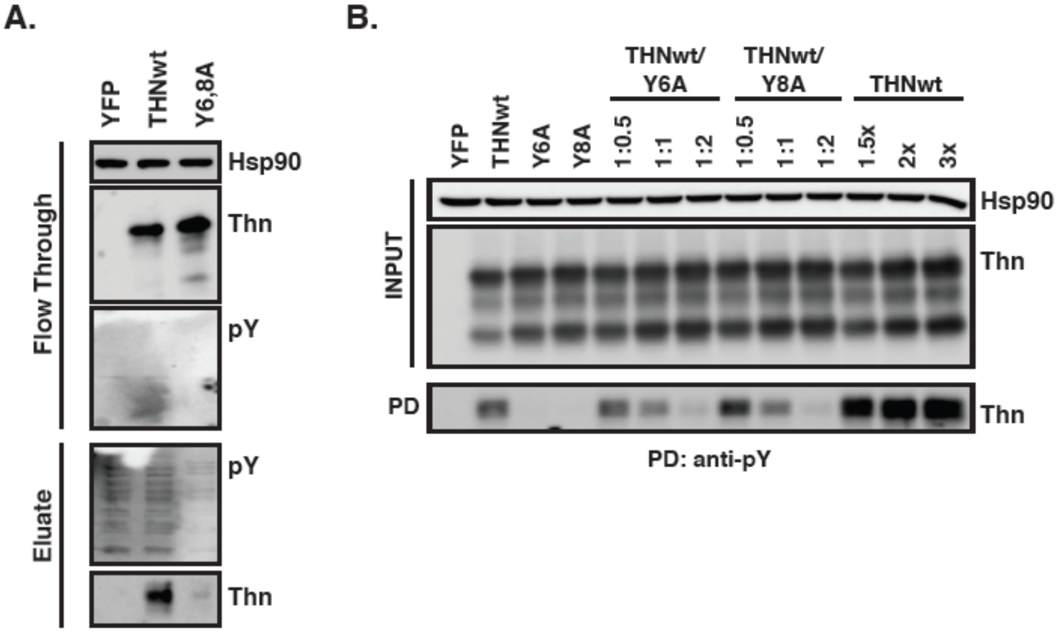

Figure S1 (related to Figure 1). Transient co-expression of tetherin mutants Y6A or Y8A have a dominant-negative effect on tyrosine phosphorylation of wild-type tetherin

(A) Phosphorylated proteins were isolated from lysates of 293 cells transiently expressing wild-type tetherin, double tyrosine mutant (Y6,8A) or YFP as control, by using a Phosphoprotein Purification Column. Phosphorylation status of the proteins was assessed by western blot analysis of the flow-through and eluated fractions. (B) Lysates from 293 cells co-transfected with a fixed amount of wild type tetherin plus increasing amounts of Y6A or Y8A, were immunoprecipitated with an anti-phosphotyrosine antibody and precipitates analyzed by western blot for the presence of tetherin.

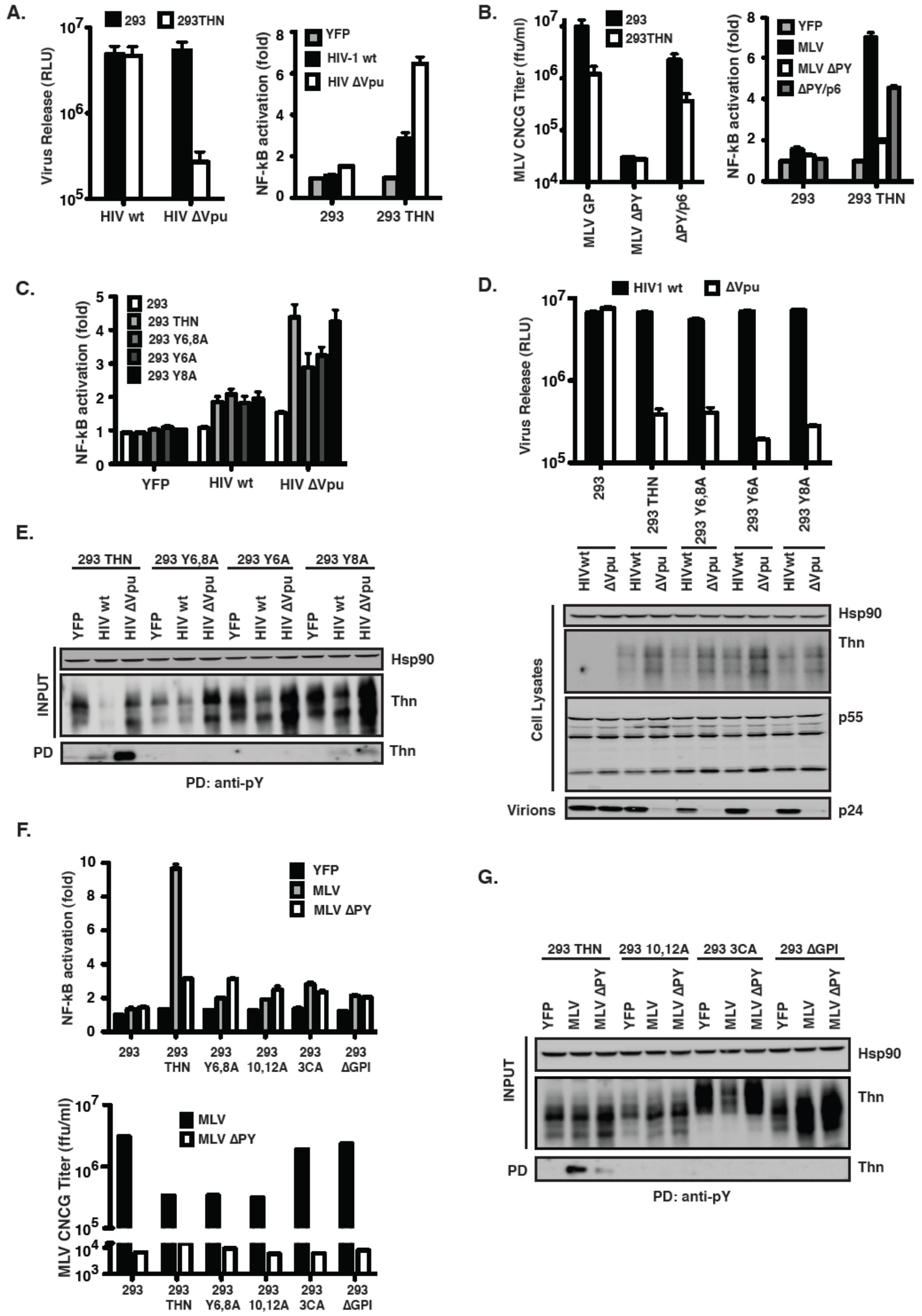

Figure S2 (related to Figure 2). Tetherin phosphorylation induced by retroviral assembly.

(A, B) Infectious virus release from supernatants of Figure 2A was determined on HeLa-TZMbl indicator cells (A, left panel), or by titration of MLV

supernatants on 293T cells and analyzed by flow cytometry for GFP expression 48h later (B, left panel). NF-kB reporter fold activation in 293 and 293/tetherin cells transfected with HIV-1 wt or HIV-1 ∆Vpu (A, right panel), or MLV provirus or derivatives (MLV∆PY, MLV∆PY/p6) (B, right panel). **(C)** NF-kB reporter fold activation in 293 cells stably expressing wild-type tetherin, the double tyrosine mutant (Y6,8A) or the single tyrosine mutants (Y6A or Y8A) transfected with HIV-1 wild-type or HIV-1 ∆Vpu proviruses. **(D)** Infectious virus release from C was determined as in (A) and physical particle yield analyzed by western blot of cell lysates and pelleted supernatants using an anti-p24 monoclonal antibody. **(E)** Lysates from (C) were used for immunoprecipitated with anti-pY antibody. Pulled-down fractions were analyzed for tetherin co-precipitation by western blot. **(F)** Stable cell lines expressing wild-type tetherin, the Y6,8A or RVP(10-12)AAA mutants or the structural mutants 3CA (cysteine-less), or ∆GPI (truncated form lacking the GPI anchor) were assayed for their ability to activate NFkB luciferase reporter upon transfection with MLV provirus or the MLV∆PY derivative (upper panel). MLV virus titers were assayed as in (B) (lower panel). **(G)** Lysates from (F) were immunprecipitated with anti-pY, deglycosylated and blotted for tetherin. NFkB luciferase reporter fold changes relative to 293 cells transfected with YFP control (A, B, C and F). All error bars represent ±SEM of three experiments.

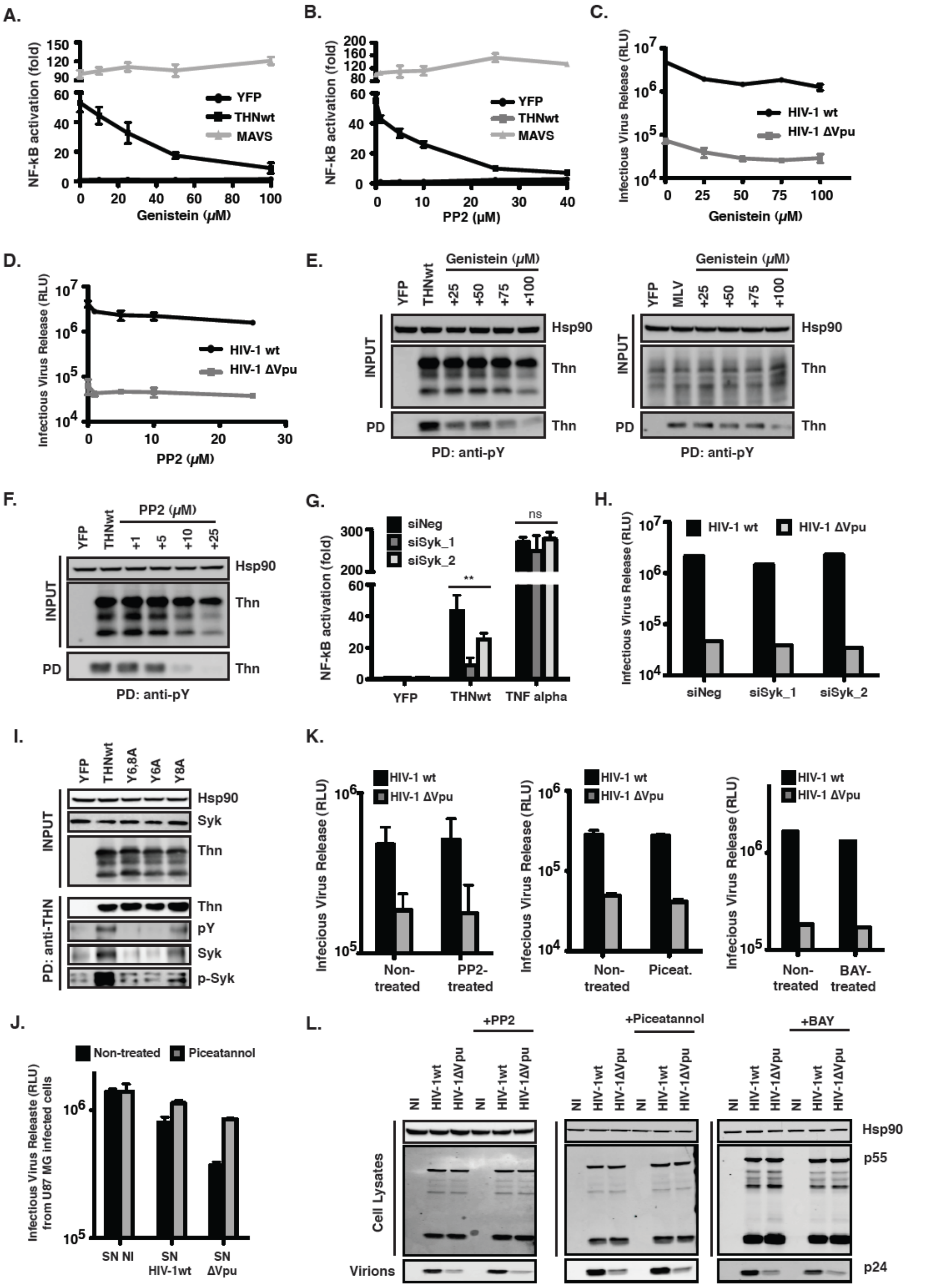

Figure S3 (related to figure 4). Treatment of cells with Tyrosine Kinase inhibitors or Syk siRNA depletion.

(A, B) NF-kB reporter fold activation in 293 cells transiently expressing tetherin, or MAVS as control, were treated with increasing amounts of the

inhibitors Genistein (A) or PP2 (B). Fold changes relative to cells transfected with YFP and non-treated with inhibitors. **(C, D)** HIV-1 Infectious viral particle release from supernatants from Figure 4A (C) and Figure 4B (D) were determined on HeLa-TZMbl indicator cells. **(E, F)** Lysates from parallel experiments to Figure S3A (E, left), Figure 4A (E, right) and Figure S3B (F) were immunoprecipitated with an anti-phosphotyrosine antibody and precipitates analyzed as before **(G)** 293 cells were transfected twice over 72h with siRNAs directed against Syk (siSyk_1 and _2) or a non-targeting control (siNeg). Cells were then transiently transfected with the NFκB reporter plasmid either with tetherin or treated with TNFα (1ng/ml). Fold increases in luciferase expression are plotted relative to YFP control. All error bars represent ±SEM of three experiments. *p > 0.05, **p > 0.01, and ***p > 0.001 as determined by paired two-tailed t test. **(H)** HIV-1 Infectious viral particle release from supernatants from Figure 4D were determined as in (C, D). **(I)** Lysates of 293 cells transiently expressing wild type tetherin or tyrosine mutants were immunoprecipitated with anti-tetherin antibodies. Pull-downs were analyzed by western blot for tetherin, phosphotyrosine, Syk and its active phosphorylated form (pSyk). **(J)** U87MG cells were treated overnight with supernatants from non-treated or Piceatannol-treated CD4+ve T cells infected in Figure 4J, and then challenged with VSV-G pseudotyped HIV-1 wt virus (MOI of 0.5) in a one-round viral replication assay. Infectious virus release was determined 48 hours later as in (C, D). **(K, L)** Infectious virus release from cells in Figure 4J was determined by infection of HeLa-TZMbl reporter cell lines (K) and western blot analysis of cell lysates and supernatants for HIV-1 p24-CA (L).

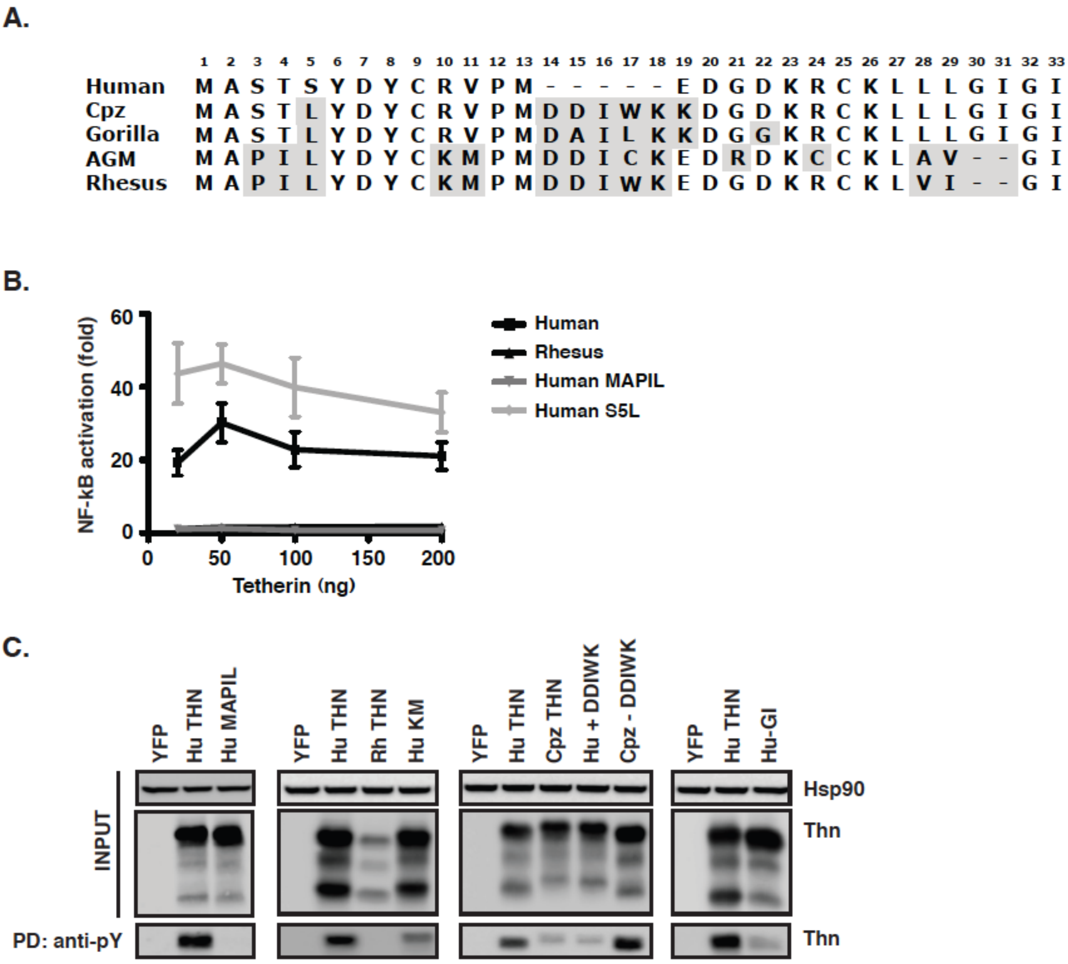

Figure S4 (related to Figure 5). Multiple context-dependent adaptations have resulted in the signaling capacity of human tetherin.

(A) Alignment of cytoplasmic tails from different primate tetherins with segments differing from human tetherin highlighted in gray. **(B)** NFkB Luciferase reporter activation in 293 cells transiently transfected with increasing amounts of human tetherins bearing rhesus-specific mutation in positions 3-4. All error bars represent ±SEM of three experiments. **(C)** Anti-pY immunoprecipitates of 293 cells transiently transfected with the indicated tetherin mutant were deglycosylated and analyzed for tetherin pulldown by western blot.

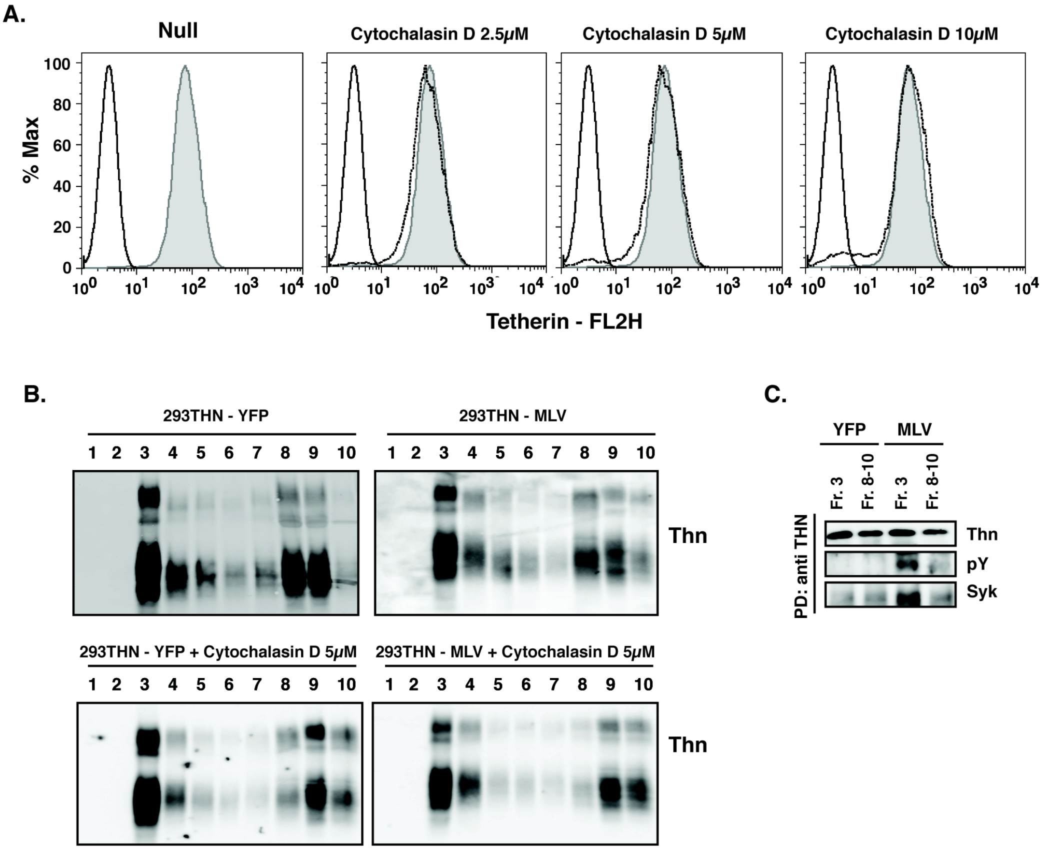

Figure S5 (related to Figure 6). Cytochalasin D treatment does not affect tetherin surface expression, and tyrosine phosphorylation of tetherin occurs preferentially in detergent resistant membranes.

(A) 293/tetherin cells were treated with increasing amounts of cytochalasin D for 8 hours and tetherin expression and the cell surface was determined by flow cytometry using anti-human tetherin-PE antibody. **(B)** 293/tetherin cells transfected with YFP or MLV provirus were mock- or cytochalasin D-treated (5μM) for 6 hours and then lysed and DRMs separated on an iodixinol gradient. Localization of tetherin was determined by western blotting the different indicated gradient fractions. **(C)** Tetherin was immunoprecipitated from DRM (fraction 3) or soluble (Fractions 8-10) fractions and pulled-downs were western blotted for tyrosine phosphorylated tetherin (anti-pY) and Syk.

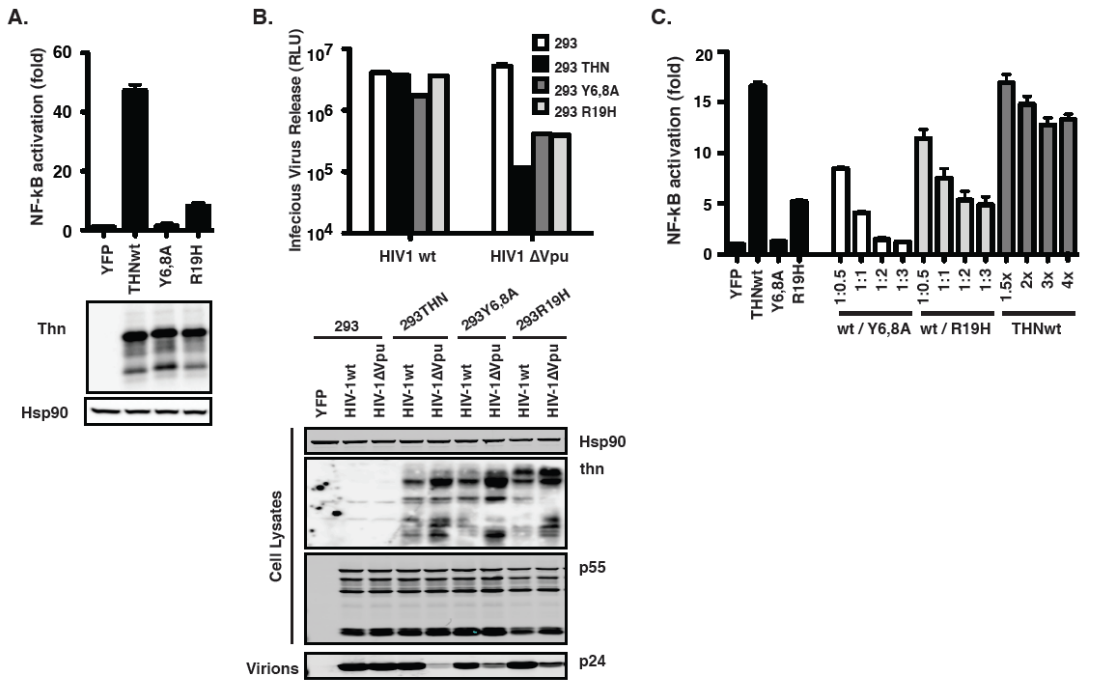

Figure S6 (related to Figure 7). Tetherin R19H polymorphism is defective for tetherin-mediated NFkB activation but not virion retention.

(A) Fold activation of a NFkB reporter in 293 cells transiently transfected with wild-type tetherin, double-tyrosine mutant (Y6,8A) or the human CT polymorphism R19H, compared to control YFP vector (upper). Tetherin expression levels were confirmed by western blot (lower). **(B)** Infectious virus release from supernatants from Figure 7B was determined on HeLa-TZMbl indicator cells (upper) and western blot analysis of cell lysates and supernatants for HIV-1 p24-CA (lower). **(C)** Fold activation of NFkB reporter in 293 cells transiently transfected with fixed amounts of NFkB luciferase reporter and wild-type tetherin plus increasing amounts of tetherin double tyrosine mutant or R19H CT polymorphism (or wild-type as internal control). Total DNA amounts transfected were kept constant by addition of pCR3.1 YFP. Fold changes relative to YFP control. Error bars represent ±SEM of 3 independent experiments.

Supplemental Experimental Procedures

Plasmids and Cells

Tetherin mutants and species orthologs were made in pCR3.1 by standard molecular biology methods. Chimpanzee and Gorilla tetherins were provided by, respectively, G. Towers (UCL) and F. Kirchhoff (Univ. of Ulm). The NFkB-dependent 3xkB-pCONA-FLuc reporter construct and the CMV-RLuc control plasmid were provided by A. MacDonald (Mankouri et al., 2010), while the MAVS expression vector was provided by J. Luban (UMASS). The molecular clones of HIV-1 NL4.3 and derived ΔVpu mutant have been described previously (Neil et al., 2006). pMLV NCS (MLV), pMLV NCS ΔPY and pMLVp6pY plasmids were provided by J. Martin-Serrano (KCL) (Martin-Serrano et al., 2004). A codon optimized RICH2 cDNA was synthesized by MWG and subcloned into pCR3.1 with an N-terminal HA tag using standard procedures. Human Embryonic Kidney 293 (HEK-293), 293T (HEK-293T) and U87-MG cells were obtained from the ATCC, while the HeLa TZM indicator cell line was obtained from Dr. John C. Kappes through the NIH AIDS Reagent Repository Program (ARRP). All adherent cells were maintained in DMEM supplemented with 10% fetal calf serum (FCS) and gentamicin.

To produce 293 transductants stably expressing tetherin mutants, the indicated mutant was cloned into the retroviral vector pLHCX (Clontech). Retroviral vector stocks were produced by cotransfection of 293T cells with MLV Gag-Pol expression vector, pMD.G (VSV-G) and pCR3.1-Vpu. 293 cells were transduced 48h later and hygromycin resistant populations were selected. Derivatives stably expressing some of the human tetherin mutants have been described previously (Galao et al., 2012).

Antibodies

Cell lysates, IPs and eluates were subjected to SDS-PAGE and western blots performed using the following antibodies: rabbit anti-BST2 (NIH ARRP), mouse anti-BST2 monoclonal (clone 3H4) (Abnova), rabbit anti-phosphotyrosine (Calbiochem), mouse anti-phosphotyrosine monoclonal (clone 4G10) (Millipore), mouse anti-HA.11 (Covance); from Cell Signaling - rabbit monoclonal anti-Syk (D1I5Q), rabbit anti-phospho Syk (Y323), rabbit anti-ZAP70, rabbit anti-phospho ZAP70 (Y292), rabbit anti-TAK1 and rabbit anti-TRAF2; from Santa Cruz Biotechnologies: rabbit anti-hsp90 α/ß, goat anti-RICH2 (S-16) and rabbit anti-TRAF6 (H-274). For HIV-1 Gag/p24, hybridoma supernatant of monoclonal ab 183 (NIH ARRP) was used at 1:100. Visualization was performed by ImageQuant using anti-mouse or anti-rabbit HRP-linked antibodies (NEB, UK) or an anti-goat-HRP linked antibody (Santa Cruz Biotechnologies), or on a LiCor imager using goat-anti mouse IRD-680/goat-anti rabbit IRD800 conjugated secondary antibodies (LiCor).

References

Galao, R.P., Le Tortorec, A., Pickering, S., Kueck, T., and Neil, S.J. (2012). Innate sensing of HIV-1 assembly by Tetherin induces NFkappaB-dependent proinflammatory responses. Cell Host Microbe *12*, 633-644.

Mankouri, J., Fragkoudis, R., Richards, K.H., Wetherill, L.F., Harris, M., Kohl, A., Elliott, R.M., and Macdonald, A. (2010). Optineurin negatively regulates the induction of IFNbeta in response to RNA virus infection. PLoS Pathog *6*, e1000778.

Martin-Serrano, J., Perez-Caballero, D., and Bieniasz, P.D. (2004). Context-dependent effects of L domains and ubiquitination on viral budding. J Virol *78*, 5554-5563.

Neil, S.J., Eastman, S.W., Jouvenet, N., and Bieniasz, P.D. (2006). HIV-1 Vpu promotes release and prevents endocytosis of nascent retrovirus particles from the plasma membrane. PLoS Pathog *2*, e39.
